# Comparison of transcriptomes from two chemosensory organs in four decapod crustaceans reveals hundreds of candidate chemoreceptor proteins

**DOI:** 10.1371/journal.pone.0230266

**Published:** 2020-03-12

**Authors:** Mihika T. Kozma, Hanh Ngo-Vu, Yuen Yan Wong, Neal S. Shukla, Shrikant D. Pawar, Adriano Senatore, Manfred Schmidt, Charles D. Derby

**Affiliations:** 1 Neuroscience Institute, Georgia State University, Atlanta, Georgia, United States of America; 2 Department of Biology, University of Toronto Mississauga, Mississauga, Ontario, Canada; 3 Department of Biology, Georgia State University, Atlanta, Georgia, United States of America; University of Missouri Columbia, UNITED STATES

## Abstract

Crustaceans express genes for at least three classes of putative chemosensory proteins. These are: Ionotropic Receptors (IRs), derived from the heterotetrameric ionotropic glutamate receptors (iGluRs); Transient Receptor Potential (TRP) channels, a diverse set of sensor-channels that include several families of chemoreceptor channels; and Gustatory Receptor Like receptors (GRLs), ionotropic receptors that are homologues of Gustatory Receptors (GRs) of insects and are expressed sparingly in most crustaceans so far studied. IRs are typically numerically the most dominant of these receptor proteins in crustaceans and include two classes: co-receptor IRs, which are necessary for making a functional receptor-channel; and tuning IRs, whose specific combination in the IR subunits in the heterotetramer confers chemical specificity. Previous work showed that the transcriptomes from two major chemosensory organs–the lateral flagellum of the antennule (LF) and the tips of the legs (dactyls)–of the Caribbean spiny lobster *Panulirus argus* express four co-receptor IRs and over 100 tuning IRs. In this paper, we examined and compared the transcriptomes from the LF and dactyls of *P*. *argus* and three other decapod crustaceans–the clawed lobster *Homarus americanus*, red swamp crayfish *Procambarus clarkii*, and the blue crab *Callinectes sapidus*. Each species has at least ca. 100 to 250 IRs, 1 to 4 GRLs, and ca. 15 TRP channels including those shown to be involved in chemoreception in other species. The IRs show different degrees of phylogenetic conservation: some are arthropod-conserved, others are pancrustacean-conserved, others appear to be crustacean-conserved, and some appear to be species-specific. Many IRs appear to be more highly expressed in the LF than dactyl. Our results show that decapod crustaceans express an abundance of genes for chemoreceptor proteins of different types, phylogenetic conservation, and expression patterns. An understanding of their functional roles awaits determining their expression patterns in individual chemosensory neurons and the central projections of those neurons.

## Introduction

Sensing environmental chemicals is critical for animals because it informs them of the presence and location of important resources. The process of chemoreception begins with an animal acquiring and detecting chemical cues. The detection is performed by chemosensory cells, whose receptor proteins bind stimulus molecules which in turn lead to a cascade of transduction events that results in activation of those cells. Our understanding of these receptor proteins has significantly evolved since the discovery by Buck and Axel in 1991 [1] that a major class of chemoreceptor proteins in the rodent olfactory system are modified rhodopsin-like type A G-protein coupled receptors (GPCR), called Odorant Receptors (OR). Since then, many more classes of chemosensory GPCRs have been discovered in mammals and other vertebrates, including trace amine-associated receptors (TAAR), vomeronasal receptor type 1 and 2 (V1R, V2R), formyl-peptide receptors (FPR), and taste receptor type 1 and 2 (T1R, T2R). Vertebrates also have a different class of important though numerically less abundant chemoreceptor proteins–ionotropic receptors–which include transient receptor potential (TRP) channels, epithelial sodium channels (ENaC), and MS4A receptors [2]

**Fig 1:**
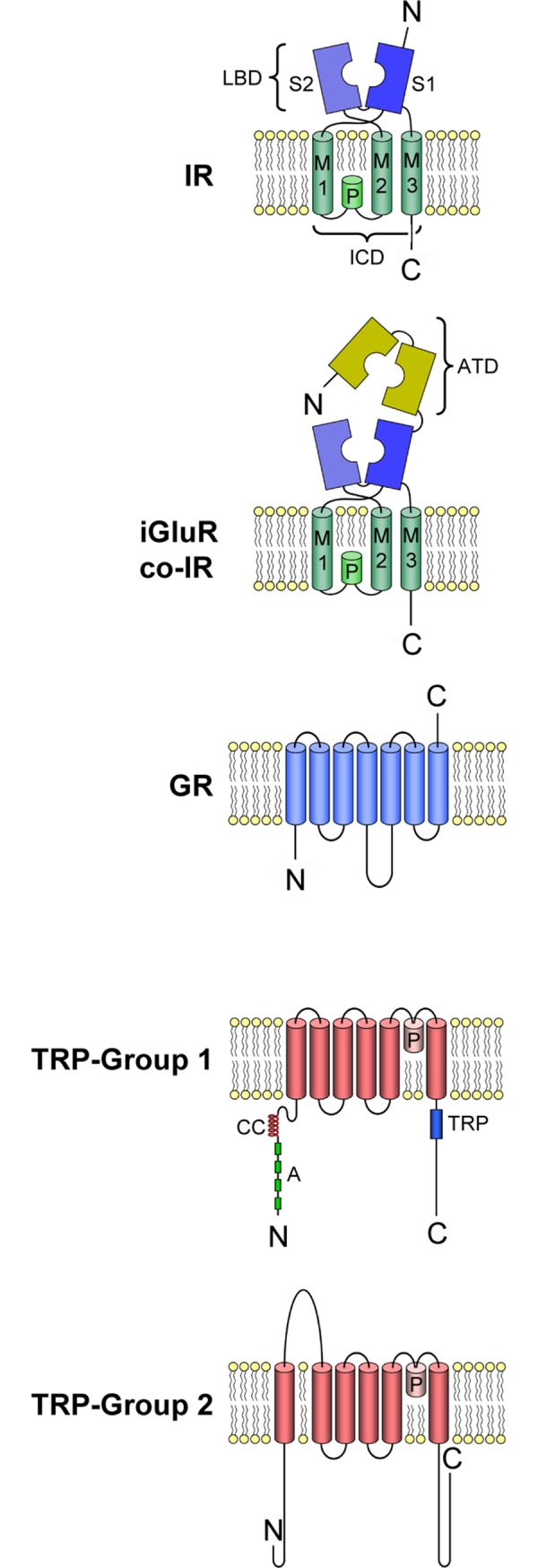
Schematic drawing of the molecular structure of putative chemoreceptor proteins co-receptor IRs and iGluRs, tuning IRs, GRs, and TRP channels. IRs, co-receptor IRs, and iGluRs contain the following domains: extracellular amino terminal domain (ATD); ion channel domain (ICD) that forms the ion channel, consisting of three transmembrane domains (M1, M2, M3) and a pore loop (P); ligand binding domain (LBD) consisting of two half-domains (S1, S2). TRP channels contain the following domains: coiled-coil domain (CC), ankyrin repeats (A), TRP domain (TRP). Adapted from [11].

Unlike the vertebrates, most metazoans including the arthropods, which are the most abundant group of animals, rely more on ionotropic receptors than GPCRs for chemical sensing [3–8]. In the major and best studied group of arthropods, the insects, ionotropic chemoreceptors include two classes of seven transmembrane ionotropic receptors called Gustatory Receptors (GRs) and Odorant Receptors (ORs), a class of three-transmembrane heterotetrameric receptors called variant Ionotropic Receptors (IRs), TRP channels, and ENaCs. Their structures with the exception of ENaCs are shown in Fig 1. GRs appear to be an ancient lineage, with GR-like receptors (GRLs) being traced possibly even to plants [4, 6, 9], though the role of GRLs in chemoreception is unknown [9]. The ORs likely evolved in insects from GRs, and so far there is no evidence of their existence in non-insect arthropods [4, 6, 10–13]. The IRs evolved from ionotropic glutamate receptors (iGluRs), and they are organized as heterotetramers [4–6] containing two classes of IRs that are different in structure and function: co-receptor IRs, for which there are four (IR25a, IR8a, IR93a, and IR76b); and tuning IRs, for which there are many more [3, 8, 14–16]. The co-receptors IR25a and IR8a differ from co-receptors IR93a and IR76b as they have high sequence identity to each other and to iGluRs. IR25a and IR8a retain the amino-terminal domain (ATD) of iGluRs that is also largely absent in most tuning IRs and have a distinctive co-receptor extra loop (CREL) region in their ligand binding domain (LBD) that is lacking in all other IRs [16]. IR25a and IR8a are also necessary for targeting the IRs to the dendritic membrane to make a functional receptor-channel and may also contribute to the tuning specificity of the cell [14]. In contrast, the roles of IR93a and IR76b as co-receptors are still ambiguous, other than being necessary for forming functional receptor channels in several chemosensory neurons. Tuning IRs, on the other hand, only confer specific sensitivity to the IRs heterotetramer by virtue of the particular combination of types. There is evidence in *Drosophila* that the heterotetrameric IR in some olfactory sensory neurons (OSNs) is composed of two co-receptor IRs and two tuning IRs [16].

The IRs have various lineages that have appeared over evolutionary history. The co-receptor IRs differ in their phylogenetic conservation [16, 17]: IR25a is “protostome conserved,” having been identified in all protostomes studied to date but not in deuterostomes or animals ancestral to the protostomes and deuterostomes. IR8a, IR93a, and IR76b are reported only in the arthropods, i.e. “arthropod-conserved” [10, 18]. The tuning IRs have evolved more recently [10, 19] and are more specific to phyla or individual species [16]. In fact, IRs are not just chemoreceptors but can participate as “environmental sensors,” detecting temperature and humidity [20–23].

Regarding other types of chemoreceptor proteins in the arthropods, the ionotropic TRP channels and ENaCs have been shown to be important in insects [3–6, 24, 25]. GPCRs have not been identified as candidates in arthropods, although pheromone transduction in the hawkmoth *Manduca sexta* may be mediated by a metabotropic signal transduction cascade [26]. On the other hand, a large family of chemosensory GPCRs are found in nematodes, and also appear to be in gastropod and cephalopod molluscs and asteroid echinoderms [4, 10, 11, 27–30].

**Fig 2:**
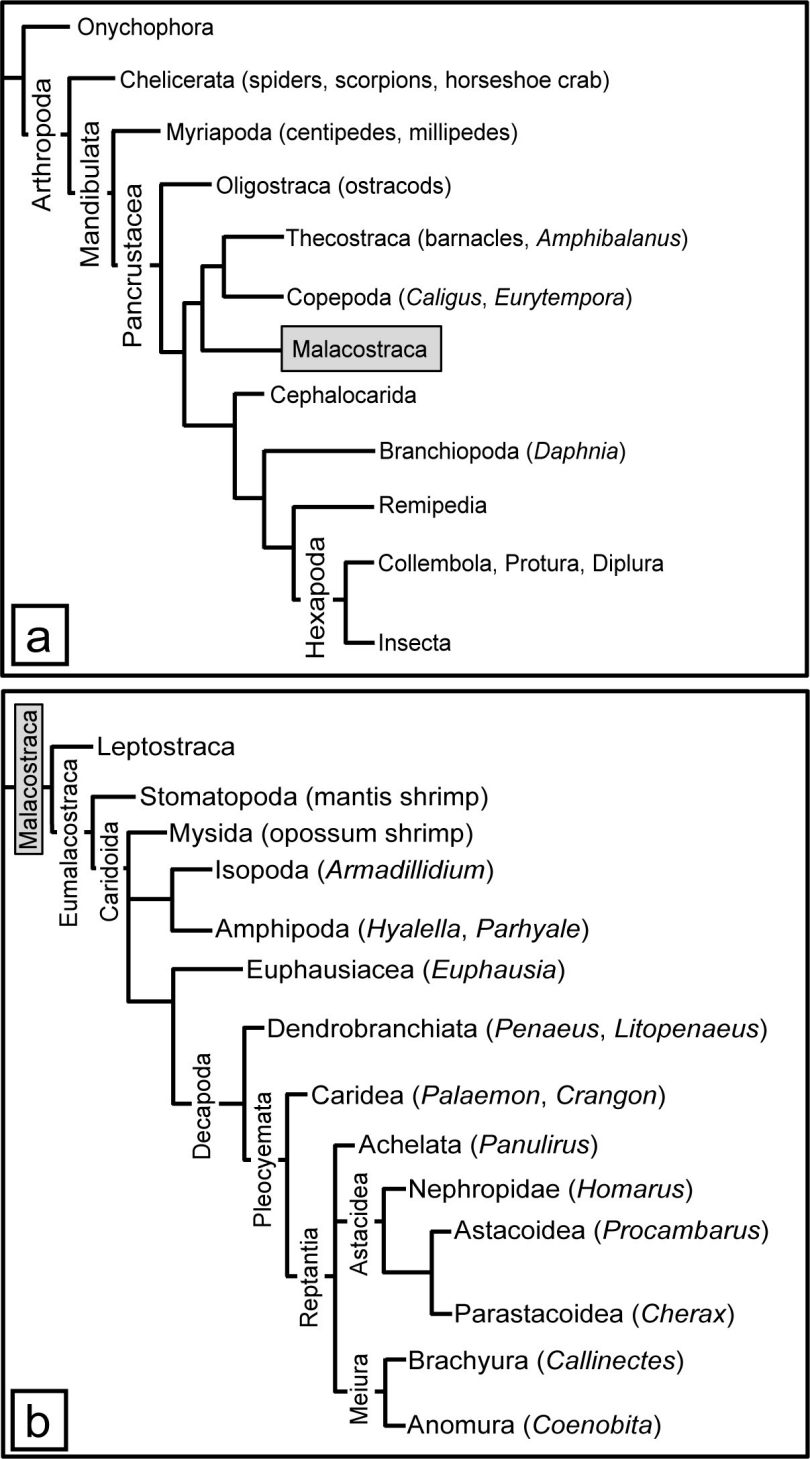
Arthropod phylogeny. Panel A shows all arthropod groups. Panel B shows an expanded view of the Malacostraca. Names of species used in our analysis are included. Based on [39–43].

Crustaceans (Fig 2) are more poorly studied regarding chemoreceptor molecules, compared to insects. Chemoreceptor expression has been examined in two chemosensory organs of the Caribbean spiny lobster, *Panulirus argus* [11]. One organ is the lateral flagellum of the antennule (LF), which mediates both olfaction (due to unimodal olfactory sensilla called aesthetascs that are innervated exclusively by OSNs) and distributed chemoreception (due to bimodal sensilla that are innervated by chemosensory neurons (CSNs) and mechanosensory neurons (MSNs)). The other chemosensory organ studied in *P*. *argus* is the walking leg dactyl, the distal-most segments of the walking legs and which mediate only distributed chemoreception [11]. *P*. *argus* and other crustaceans appear to rely most heavily on IRs for chemoreception, a claim that is based solely on IRs being found in chemosensory organs of all species examined and with the co-receptor IR25a being expressed in most or all OSNs and CSNs [11, 31–36]. Besides IRs, GRs are also prevalent in at least some crustaceans, though no ORs are reported [8, 37]. For example, the amphipod *Hyalella azteca* has 155 GRs, the branchiopod *Daphnia pulex* has 59 GRs, and the copepod *Eurytempora affinis* has 67 GRs [18]. Other crustaceans have been reported to have very limited GR representation [10, 11, 38]. Homologues of insect chemoreceptive TRP channels have also been identified in crustaceans [11]. Many IRs, one GRL, and representatives from each of the major types of TRP channels were found in *P*. *argus* [11].

The goal of the current study is to extend our analysis of chemoreceptor proteins of decapod crustaceans by studying three additional decapod species commonly used in studies of chemoreception: American lobster *Homarus americanus*, crayfish *Procambarus clarkii*, and blue crab *Callinectes sapidus*. These decapod species were chosen for their phylogeny (Fig 2), habitat, and lifestyle. *H*. *americanus* is similar to *P*. *argus* with its “lobster”-like body form, long life span, and life in a complex marine environment, but it is phylogenetically distant from *P*. *argus*. Its use of chemoreception in feeding, social behavior, and sexual behavior is well described [44, 45]. *P*. *clarkii* is phylogenetically close to *H*. *americanus*, but unlike the lobster it is a freshwater crustacean with a shorter lifespan. Its chemical senses and chemical sensing are also well described [46–53]. *C*. *sapidus* is a brachyuran crab, phylogenetically most dissimilar from the other three decapods, with a euryhaline life that allows it to live in both marine and essentially freshwater environments. Its chemoreception has been studied with respect to feeding [54–56], and sexual behavior and pheromones [57–59]. Our goal was to describe the diversity and phylogenic relationship of the chemoreceptor proteins of these decapods relative to other arthropods. We discovered a broad expansion of variant IRs, TRP channels from all subfamilies, and a few GRLs in the chemosensory organs of these four decapod crustaceans.

## Materials and methods

### Animals

Male and female Caribbean spiny lobsters, *Panulirus argus*, American lobsters, *Homarus americanus*, red swamp crayfish, *Procambarus clarkii*, and blue crabs, *Callinectes sapidus* were used. Specimens of spiny lobsters were collected in the Florida Keys and kindly provided by the Florida Keys Marine Laboratory and Dr. Donald Behringer (University of Florida). Specimens of *H*. *americanus*, *P*. *clarkii*, and *C*. *sapidus* were obtained from H Mart (Doraville, GA), with *H*. *americanus* having been collected in New England, *P*. *clarkii* in Louisiana, and *C*. *sapidus* in the U.S. southeast coast. Animals were held at Georgia State University in communal 800-L aquaria or in individual 10-L aquaria containing aerated, recirculated, filtered artificial seawater (Instant Ocean, Aquarium Systems, Mentor, OH) for the marine species and dechlorinated tap water for *P*. *clarkii* in a 12-hr:12-hr light:dark cycle. They were fed shrimp or squid three times per week.

### Tissue collection and RNA isolation for generating transcriptomes

Animals was anesthetized on ice prior to tissue collection. For *P*. *argus*, tissues were collected from four adult animals (three females and one male), as described in Kozma et al. (2018) [11]. For *H*. *americanus*, tissues were collected from an adult male (weight 484 g, carapace length 114 mm) and an adult female (weight 463 g, carapace length 109 mm). For *P*. *clarkii*, nine animals were used (five adult females and four adult males, mean weight 37.1 g and mean carapace length 36.5 mm), with five animals being used for extracting RNA from antennules, two for dactyls (one from each sex), and two for brains (one from each sex). For *C*. *sapidus*, ten animals (eight adult males and two adult females, mean weight 161.5 g, mean carapace length 143 mm) were used for extracting RNA from antennules, and one male and one female from the same group of animals were used for dactyl tissue extraction.

Three tissues were collected: aesthetasc-bearing region of both antennular lateral flagella (LF); sensilla-bearing dactyl of the second walking legs (dactyl); and supraesophageal ganglion (brain) (only in *P*. *argus*, *H*. *americanus*, and *P*. *clarkii*).

In *P*. *argus* and *H*. *americanus*, the soft tissue within the cuticle of LF and dactyls was dissected out from each animal and pooled together according to tissue type for each species. In *P*. *clarkii* and *C*. *sapidus*, the soft tissue of the dactyl was dissected out of the cuticle, while the LFs were collected with the tissue still within the cuticle due to their small size. Cuticle was removed by homogenizing tissue, centrifugation, and filtration of supernatant prior to RNA extraction. Collected tissues were frozen instantly in liquid nitrogen and stored at -80^o^ C until RNA extraction [11].

To extract total RNA, the same methods as described in Kozma et al. (2018) [11] were used. Frozen tissues were homogenized in Tri-Reagent (Sigma-Aldrich, St. Louis, Missouri). Sequential centrifugation with chloroform and ethanol was used to precipitate RNA from the tissue. DNA, protein, and carbohydrate contaminants were removed by reconstituting RNA in diethylpyrocarbonate (DEPC)-treated water and then precipitating again in lithium chloride. Potassium acetate was used to precipitate other possible contaminants such as sodium dodecyl sulfate (SDS) and SDS-bound proteins and leave RNA in solution, which was then precipitated out of solution using ethanol. Total RNA reconstituted in DEPC-treated water was tested for concentration and purity using NanoDrop 2000c spectrophotometer (Thermo Fisher Scientific, Waltham, Massachusetts). Total RNA extracted for each tissue type of each species was frozen over liquid nitrogen and stored in aliquots at –80°C.

### RNA sequencing, *de novo* assembly, and transcript abundance estimation

Quality assessment on Agilent Bioanalyzer2000 and TapeStation of total RNA extracted, mRNA specific cDNA synthesis, and cDNA paired-end sequencing on the Illumina HiSeq 2500 high-throughput sequencer were performed by Beckman Coulter Genomics (now part of GENEWIZ, South Plainfield, New Jersey) similar to Kozma et al. (2018) [11]. For *P*. *argus*, the read length was 2x100 (base pair reads) for LF and dactyl, and 2x125 (base pair reads) for brain. For *H*. *americanus*, the read length was 2x125 (base pair reads) for all three tissues. For *P*. *clarkii*, the read length was 2x125 (base pair reads) for LF and dactyl and 2x100 (base pair reads) for brain. For *C*. *sapidus*, the read length was 2x125 (base pair reads) for both tissues. The number of reads per sample was > 120 million. Adapter sequences tracking Illumina reads from multiplexed samples were removed prior to delivery. The reads were deposited to NCBI under BioProject accession PRJNA596786, with SRA accessions SRR10874089, SRR10874088, SRR10874086, SRR10874085, SRR10874084, SRR10874083, SRR10874082, SRR10874081, SRR10874080, SRR10874079, and SRR10874087. Raw reads for each species were concatenated prior to transcriptome assembly. For example, for *P*. *argus*, left reads from LF, dactyl, and brain were concatenated into one file, and right reads were concatenated into one file. Eight independent *de novo* assemblies were first generated for each species using different transcriptome assembly software, as described below. A reference transcriptome for each species was then generated by using the EvidentialGene pipeline (https://f1000research.com/posters/5-1695).

#### Trinity *de novo* assemblies

*Unnormalized Trinity de novo Assembly*. The first *de novo* transcriptome was assembled via Trinity v.4.0 [60]. Raw reads were compiled into left and right read files respectively, and processed through Trinity-Trimmomatic v.4.0 [60, 61] for trimming of 3’-ends of the sequenced reads. This *de novo* transcriptome was generated without normalization of reads.

*Normalized Trinity de novo Assembly*. The second transcriptome was then assembled with Trinity v.4.0 [60]. Raw reads were compiled into left and right read files respectively, and processed through Trinity-Trimmomatic v.4.0 [60, 61] for trimming of 3’-ends of the sequenced reads. This *de novo* transcriptome was generated with the default Trinity v2.4.0 normalization of reads.

#### Other *de novo* assemblies

Normalization: The remaining six transcriptomes were all generated using normalized reads. The trimmed raw reads (processed through Trinity-Trimmomatic v.4.0, obtained from the trimming process of the first transcriptome assembly mentioned above) were normalized with FastUniq [62] to remove redundancy in reads data.

*Normalized TransAbyss de novo Assemblies*. The third, fourth, and fifth transcriptomes were generated using Trans-Abyss v1.5.3 [63], with K-mer sizes 63, 87, and 111, respectively.

*Normalized Velvet/OASES de novo Assemblies*. The sixth, seventh, and eighth transcriptomes were generated using Velvet v1.2.10 and OASES v0.2.09, with K-mer-sizes 63, 87, and 111, respectively.

#### EvidentialGene pipeline

All eight transcriptome assemblies for each species were input to EvidentialGene (EVG) pipeline to give a single refined transcriptome for each species with transcript and protein-coding gene counts shown in S1 Table.

The TransDecoder program (http://transdecoder.github.io/) was used to predict protein sequences from transcripts based on open reading frames (ORF). CD-Hit [64] was performed on each transcriptome to remove redundancy. All further analyses were performed on the cd90 datasets generated by CD-Hit. BUSCO v3 was run on the transcriptomes to analyze the completeness of the assembly using the *Arthropoda odb9* lineage (Creation date: 2017-02-07, number of species: 60, number of BUSCOs: 1066) [65–67]. The BUSCO output for *P*. *argus* was **C**:91.7% [**S**:89.1%, **D**:2.6%], **F**:1.0%, **M**:7.3%, **n**:1066; for *H*. *americanus*, **C**:91.5% [**S**:89.5%, **D**:2.0%], **F**:1.0%, **M**:7.5%, **n**:1066; for *P*. *clarkii*, **C**:92.8% [**S**:90.0%, **D**:2.8%], **F**:0.9%, **M**:6.3%, **n**:1066; for *C*. *sapidus*, **C**:96.2% [**S**:85.8%, **D**:10.4%], **F**:1.3%, **M**:2.5%, **n**:1066; where **C** = complete BUSCOs, **S** = complete and single-copy BUSCOs, **D** = complete and duplicated BUSCOs, **F** = fragmented BUSCOs, **M** = missing BUSCOs, and **n** = total BUSCO groups searched (S2 Table).

Following the removal of redundancy, the abundance of transcripts for each transcriptome was estimated using RSEM [68] bundled into the Trinity v2.8.2 package for each tissue type and a counts matrix was generated. Custom ‘R’ script (S1 File) for DESeq2 [69] was used to measure fold differences of transcript abundance within each transcriptome to predict tissue specificity for a given transcript of interest. Transcripts whose expression was log_2_[fold change] ≥ 1.5 or log_2_[fold change] ≤ –1.5, according to DESeq2 (~ 2.8 fold actual change) were considered to have a higher level of expression in one tissue compared to the other (i.e. LF vs. dactyl). Transcripts whose fold change was in between this range were considered to be expressed at the same level in both tissues.

#### IR identification, sequence alignment, and phylogenetic analysis

Screening for IRs and iGluRs was performed with TMHMM v2.0 for transmembrane domain prediction and domain region screened with InterProScan 5 (v5.28–67.0) [70] for conserved Pfam [71] and InterPro [72] domains on high performance computing systems at Georgia State University [73, 74]. IRs have several distinctive domains: an extracellular amino-terminal domain (ATD) involved in assembly of the heteromeric channel; an extracellular ligand binding domain (LBD) consisting of two half-domains (S1 and S2) to which agonists bind; an ion channel domain (ICD) that forms the ion channel, consisting of three transmembrane domains (M1, M2, M3) and a pore loop (P); and an intracellular carboxyl-termination domain (CTD). The ICD domain and S2 of the LBD were predicted by the presence of the Pfam domain PF00060 (which contains M1, P, M2, S2, and M3). S1 of the LBD was predicted by the presence of the Pfam domain PF10613. All predicted protein sequences from the transcriptomes that contained both PF00060 and PF10613 domain regions were considered putative IRs and selected for phylogenetic analyses. IRs and iGluRs from *P*. *argus* that were previously identified [11] were used as reference sequences. In *P*. *argus*, following transcriptome assembly with the EvidentialGene pipeline, we identified more complete putative IR sequences than predicted previously [11]. Phylogenetic trees were built as described in Kozma et al. (2018) [11]. Selected sequences were aligned using default settings for MAFFT [75, 76]. Alignments were visualized and trimmed on Jalview [77] to remove gaps and regions of aligned sequences with low amino acid conservation. Sequences that had large gaps in the LBD and ICD regions were removed, with some exceptions. IQ-Tree [78, 79] was used for constructing maximum likelihood phylogenetic trees, along with ModelFinder [80] integrated into IQ-Tree to automatically determine the best model of substitution (see selected models in figure legends). Ultrafast bootstrap (UFBoot) [81] integrated into IQ-Tree was used to generate confidence values for the trees. The phylogenetic trees were visualized using FigTree v1.4.2 (http://tree.bio.ed.ac.uk/software/figtree/), and color schemes were edited on Adobe Illustrator CS6, San Jose, CA.

Phylogenetic analysis of conserved IRs also included conserved IR sequences from several arthropod species (S12 Table). These include chelicerates [horseshoe crab *Limulus polyphemus* (Lpol), mites [*Galendromus occidentalis* (Gocc), *Tetranychus urticae* (Turt), *Leptotrombidium deliense* (Ldel), *Dinothrombium tinctorium* (Dtin), and *Tropilaelaps mercedesae* (Tmer)], tick *Ixodes scapularis* (Isca), scorpion *Centruroides sculpturatus* (Cscu), spider *Parasteatoda tepidariorum* (Ptep)]; myriapod [*Strigamia maritima* (Smar)]; crustaceans [thecostracan barnacle *Amphibalanus improvisus* (Aimp), copepod *Eurytemora affinis* (Eaff), isopod *Armadillidium vulgare* (Avul), amphipod *Hyalella azteca* (Hazt), decapod *Litopenaeus vannamei* (Lvan), branchiopod *Daphnia pulex* (Dpul)]; insects [*Drosophila melanogaster* (Dmel), *Aedes aegypti* (Aaeg), *Culex quinquefasciatus* (Cqui), *Anopheles gambiae* (Agam), *Bombyx mori* (Bmor), *Tribolium castaneum* (Tcas), *Apis mellifera* (Amel), *Nasonia vitripennis* (Nvit), *Acrythosiphon pisum* (Apis), and *Pediculus humanus humanus* (Phum)]; and two gastropod molluscs [*Aplysia californica* (Acal) and *Lottia gigantea* (Lgig)].

### IR and iGluR nomenclature

IRs and iGluRs were named as previously described in Kozma et al. (2018) [11]. Sequences from each species were given one of the following prefixes: Parg (*P*. *argus*), Hame (*H*. *americanus*), Pcla (*P*. *clarkii*), or Csap (*C*. *sapidus*). Newly identified Parg IR sequences from the EvidentialGene pipeline were arbitrarily assigned numbers increasing from 1095 following the nomenclature in Kozma et al. 2018 [11], e.g. PargIR1095, PargIR1096, and so on. Some newly identified sequences were named by assigning suffixes “b”, “c,” and on so to previously assigned sequence numbers in order to maintain continuity of numbers within a cluster of closely related IRs. Sequences were given numbers increasing from 2000 for *H*. *americanus*, 3000 for *P*. *clarkii*, and 4000 for *C*. *sapidus*. Sequences from Hame, Pcla, and Csap with homologues to Parg sequences were given the same number as the Parg sequence. Sequences from Pcla and Csap with homologues to only Hame sequences were given the same number as the Hame sequence. Sequences from Csap with homologues to only Pcla sequences were given the same number as the Pcla sequence. Whenever there were multiple homologues to a sequence in one species, suffixes “a”, “b,” and on so on were attached to each homologue. Therefore, only conserved IRs across the four decapod species share the same numbers. NMDA iGluRs were named according to their homologues in other species. Non-NMDA iGluRs were assigned arbitrary numbers, where the same number across species indicates homologues (S12 Table).

### GRL identification and sequence alignment

The transcriptomes were screened using InterProScan for the Pfam domain family, 7tm_7 (PF08395), since this family includes GRs and ORs found in insects. Randomly selected GRs from *Drosophila melanogaster*, *Daphnia pulex*, and *Eurytemora affinis* were used as reference sequences for multiple sequence alignment using MAFFT and visualized on Jalview. GRL numbers for each species do not indicate that they are homologues across other species (e.g. PargGR1 and HameGR1 are not homologous).

### TRP channels identification

Similar to putative IR and GR sequences, TRP channels were identified using InterProScan and screening for Pfam domain regions that are typically found in the different subfamilies of TRP channels: PF06011, PF08344, PF00520, PF12796, PF00023, PF16519, and PF08016. Multiple sequence alignments were constructed using MAFFT. Reference TRP channel sequences included in the alignments were from *D*. *melanogaster*, *B*. *mori*, *T*. *castaneum*, *A*. *mellifera*, *N*. *vitripennis*, *P*. *humanus humanus*, *D*. *pulex*, *Caenorhabditis elegans*, *Rattus norvegicus*, and *Mus musculus*. Maximum likelihood trees were constructed using IQ-Tree with confidence values generated by 1000 bootstraps using UFBoot, where the model of substitution was predicted by ModelFinder and shown in the figure legend. The trees were visualized on FigTree v.1.4, and color schemes were edited on Adobe Illustrator.

### Immunocytochemistry

LF and dactyls of second and third pereiopods of male and female *P*. *argus* (carapace length 34–65 mm, weight 45–250 g, n = 6) and of male and female *H*. *americanus* (carapace length 109–122 mm, weight 458–569 g, n = 4) were dissected after anesthetizing the animals on ice for about 20 min. LF were cut into 8-annuli long pieces as described previously [82], and dactyls were cut into 2 or 3 pieces. Tissue was fixed for 6–24 hr at room temperature in 4% paraformaldehyde in 0.1 M Sörensen phosphate buffer (SPB) containing 15% sucrose. Tissue was then decalcified by incubation in 10% EDTA in SPB for about one week (pieces of LF) or 2 weeks (pieces of dactyls) with several changes of the medium and then stored in 0.02 M SPB with 0.02% sodium azide at 4^o^ C. For sectioning, tissues were embedded in 300-bloom gelatin with some modifications to method described in detail previously [82]. According to this procedure, tissue pieces were first incubated for at least 1 hr in warm (60° C) gelatin to facilitate penetration of the entire internal tissue (according to Long (2018) [83]), then pieces were embedded in warm gelatin in a small disposable paraffin mold, the gelatin was hardened by cooling it on ice, and finally the gelatin was hardened by fixing it with 4% paraformaldehyde at 4^o^ C overnight. Tissue pieces in hardened gelatin were cut on a vibrating microtome (VT 1000 S; Leica, Wetzlar, Germany) into 80–100 μm thick sagittal sections.

Free-floating sections were incubated overnight at room temperature with an affinity-purified polyclonal rabbit antiserum against IR25a of *H*. *americanus* (anti-HaIR25a - courtesy of Dr. Timothy McClintock, University of Kentucky) diluted 1:750 in SPB containing 0.3% Triton-X-100 (TSPB). Anti-HaIR25a (previously annotated as anti-GluR1) was generated using two non-overlapping peptides (P1_Ha_: TGEGFDIAPVANPW; P2_Ha_: REYPTNDVDKTNFN) from the C-terminus of *H*. *americanus* IR25a (originally annotated as OET-07; Genbank accession #AY098942) [31, 32]. Sequence alignments of P1 and P2 with the deduced amino acid sequences of all IRs and iGluRs of *P*. *argus* identified in our transcriptome sequencing project showed close matches for both peptides only in *P*. *argus* IR25a, with P1_Ha_ at 79% identity and P2_Ha_ at 86% identity. For *P*. *clarkii*, P1_Ha_ = 57% identity and P2_Ha_ = 43% identity, and for *C*. *sapidus*, P1_Ha_ = 71% identity and P2_Ha_ = 36% identity. Consequently, the anti-HaIR25a yielded results for *H*. *americanus* and *P*. *argus* but not *P*. *clarkii* or *C*. *sapidus*.

Anti-HaIR25a was combined with a mixture of two mouse monoclonal antibodies against modified α-tubulin isoforms that are enriched in neurons [84] to achieve labeling of all sensory neurons. These tubulin antibodies were anti-tyrosine tubulin (T9028, clone TUB-1A2, Sigma-Aldrich, St. Louis, Missouri) diluted 1:2000 and anti-acetylated tubulin (sc-23950, Santa Cruz Biotechnology, Dallas, Texas) diluted 1:200. Our previous immunocytochemical studies on *P*. *argus* [11] showed that this mixture of anti-tubulin labeled all bipolar sensory neurons in the LF, dactyl, and second antennae, and that while epithelial cells and the walls of hemolymph vessels could also be labeled, these cells are easily distinguishable from sensory neurons based on location and morphology.

After incubation in primary antibodies, sections were rinsed 4 x 30 min in TSPB and then incubated in a mixture of two secondary antibodies, goat anti-rabbit CY3 (111-165-003, Jackson ImmunoResearch, West Grove, Pennsylvania) diluted 1:400 and goat anti-mouse DyLight-488 (35502, Thermo Fisher Scientific, Waltham, Massachusetts) diluted 1:100 in TSPB. After rinsing 3 x 30 min in TSPB, sections were incubated for 20 min in Hoechst 33258 diluted 1:150 in TSPB from a stock solution of 1 mg/ml to stain nuclei. After a final rinse in SPB, sections were mounted on slides in 1:1 glycerol:SPB containing 5% DABCO (diazabicyclol[2.2.2]octane) to prevent photobleaching. Coverslips were secured with nail polish, and slides were stored at 4^o^ C or at –20^o^ C for extended storage time.

Labeled sections were viewed and imaged at low magnification in an epifluorescence microscope equipped with color CCD camera (AxioScope FL LED with Axiocam 503, Carl Zeiss Microscopy, Thornwood, New York) and imaged at higher magnification in a confocal microscope (LSM 700, Carl Zeiss Microscopy) using the associated software package ZEN. Stacks of optical sections each 0.3–1.0 μm thickness covering from several μm to the entire section thickness of 80 μm were collected. LSM Image Browser software (version 4.2.0.121, Carl Zeiss MicroImaging GmbH, Jena, Germany) was used to select sub-stacks of optical sections (0.5–3.0 μm total thickness) and collapse them to two-dimensional images using maximum-intensity projection. PaintShopPro 6 (Jasc Software, Eden Prairie, Minnesota) was used to optimize brightness and contrast of the images and to filter out pixel noise. Final image plates were assembled in Adobe Illustrator.

**Fig 3:**
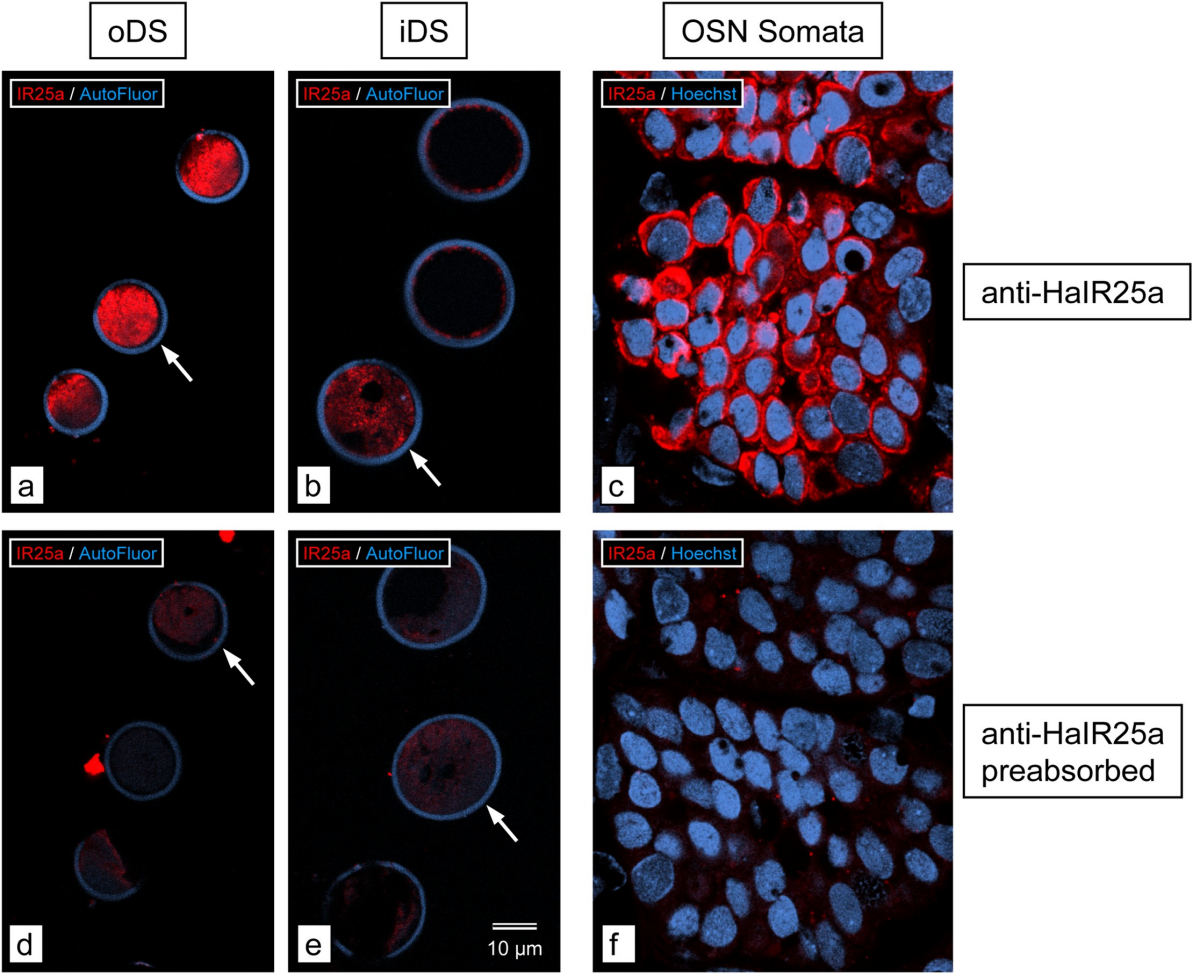
Pre-absorption control for anti-HaIR25a. Cross sections through aesthetascs of *P*. *argus*; single optical sections of 0.5 μm thickness. **a–c.** Sections labeled with control anti-HaIR25a (red) and Hoechst 33258 (blue). **d–f.** Sections labeled with preabsorbed (with P1_Pa_ and P2_Pa_) anti-HaIR25a (red) and Hoechst 33258 (blue). Scale bar in **e** applies to all images. Arrows in **a**, **b**, **d**, and **e** point to cross-sections of aesthetascs in which anti-HaIR25a labeling is captured at the very surface of the section (highest intensity). Images **a**, **b**, **d**, and **e** were collected with the same intensity setting of the red fluorescence channel of the confocal microscope; images **c** and **f** were collected at a higher (but between **c** and **f** consistent) intensity setting to compensate for the fact that labeling intensity of anti-HaIR25a is much higher in the inner dendritic segments (iDS) and outer dendritic segments (oDS) of OSNs than in the somata as was previously reported [11]. Labeling intensity is high in (**a**) oDS, (**b**) iDS, and (**c**) somata of OSNs with the control anti-HaIR25a, but below detectability in (**d**) oDS), (**e**) iDS, and (**f**) somata of OSNs with preabsorbed anti-HaIR25a, demonstrating the specificity of anti-HaIR25a for IR25a in *P*. *argus* in addition to that demonstrated by Stepanyan et al. [32] for IR25a in *H*. *americanus*.

To scrutinize the specificity of anti-HaIR25a to label IR25a of *P*. *argus*, we did a pre-absorption control using the corresponding *P*. *argus* IR25a peptides (P1_Pa_: GGDGYDIAPVANPW; P2_Pa_: REYPTNDVDKSNFT). We incubated 20 μl of anti-HaIR25a with 1 mg of each peptide in 800 μl SPB at 4°C overnight (according to Stepanyan et al. 2004 [32]) and in parallel prepared a control antibody (20 μl anti-HaIR25a in 800 μl SPB at 4°C overnight). Then we used the pre-absorbed and control antibodies at 1:750 final dilution in TSPB to label alternating 50 μm-thick vibratome sections through an 8-annuli long section of distal (aesthetasc-bearing) LF of *P*. *argus*. Confocal images of sections labeled with pre-absorbed anti-HaIR25a show a lack of specific labeling in all parts of OSNs (outer dendritic segments: oDS, inner dendritic segments: iDS, and somata), while adjacent sections labeled with control anti-HaIR25a (and collected at the same intensity setting of the confocal channel for CY3) show specific labeling in all parts of OSNs (Fig 3). As reported previously [11], oDS were labeled with highest intensity, followed by iDS, and then OSN somata. The result of the pre-absorption control confirms that anti-HaIR25a binds to and therefore labels *P*. *argus* IR25a. In general, anti-HaIr25a has some limits in its ability to penetrate tissue, so it is most effective in labeling cells on the surface of the tissue.

## Results

We previously identified IRs, GRLs, and TRP channels expressed in two chemosensory organs–LF and dactyl–of the Caribbean spiny lobster, *P*. *argus*, based on sequence homology to receptors in other species [11]. In this paper, we expanded our analysis to include three additional species: *H*. *americanus*, *P*. *clarkii*, and *C*. *sapidus*. We used the EvidentialGene pipeline to generate a single refined *de novo* transcriptome for each species, using reads generated from RNA-Seq of two chemosensory organs (LF and dactyl) for each species and brain for three species (*P*. *argus*, *H*. *americanus*, and *P*. *clarkii*). Furthermore, we estimated the abundance of transcripts for each transcriptome using RSEM and report the fold differences of abundance (LF vs. dactyl) to predict tissue specificity for transcripts of interest. Our findings are described in the following sections according to receptor type.

### IRs

The total numbers of sequences having the PF00060 domain (consisting of M1, P, M2, S2, and M3 region: see Fig 1), PF10613 domain (consisting of the S1 region), or both domains of iGluR and IR for the transcriptomes (generated from LF, dactyl, and brain) for each of the four species are shown in Table 1. All sequences containing PF00060 and PF10613, respectively, are in S2–S9 Files. Using only sequences with both domains generates a conservative estimate of the number of iGluRs and IRs, and these values range from 96 for *P*. *clarkii* to 252 for *P*. *argus*. The estimated numbers of IRs are much higher when based on sequences having only one of the two domains.

**Table 1:** Number of predicted IRs and iGluRs in transcriptomes of four decapod crustacean species, based on either or both PF domains.

Species	PF00060	PF10613	Both
***Panulirus argus***	463	375	252
***Homarus americanus***	259	200	183
***Procambarus clarkii***	181	134	96
Callinectes sapidus*	253	198	184

Number of sequences that have the respective domain region represented in the columns for each of the four decapod crustaceans. “Both” shows the number of sequences that have both PF00060 and PF10613 domain regions.

* indicates that transcriptome only has LF and dactyl tissue, while the others have LF, dactyl, and brain.

**Fig 4:**
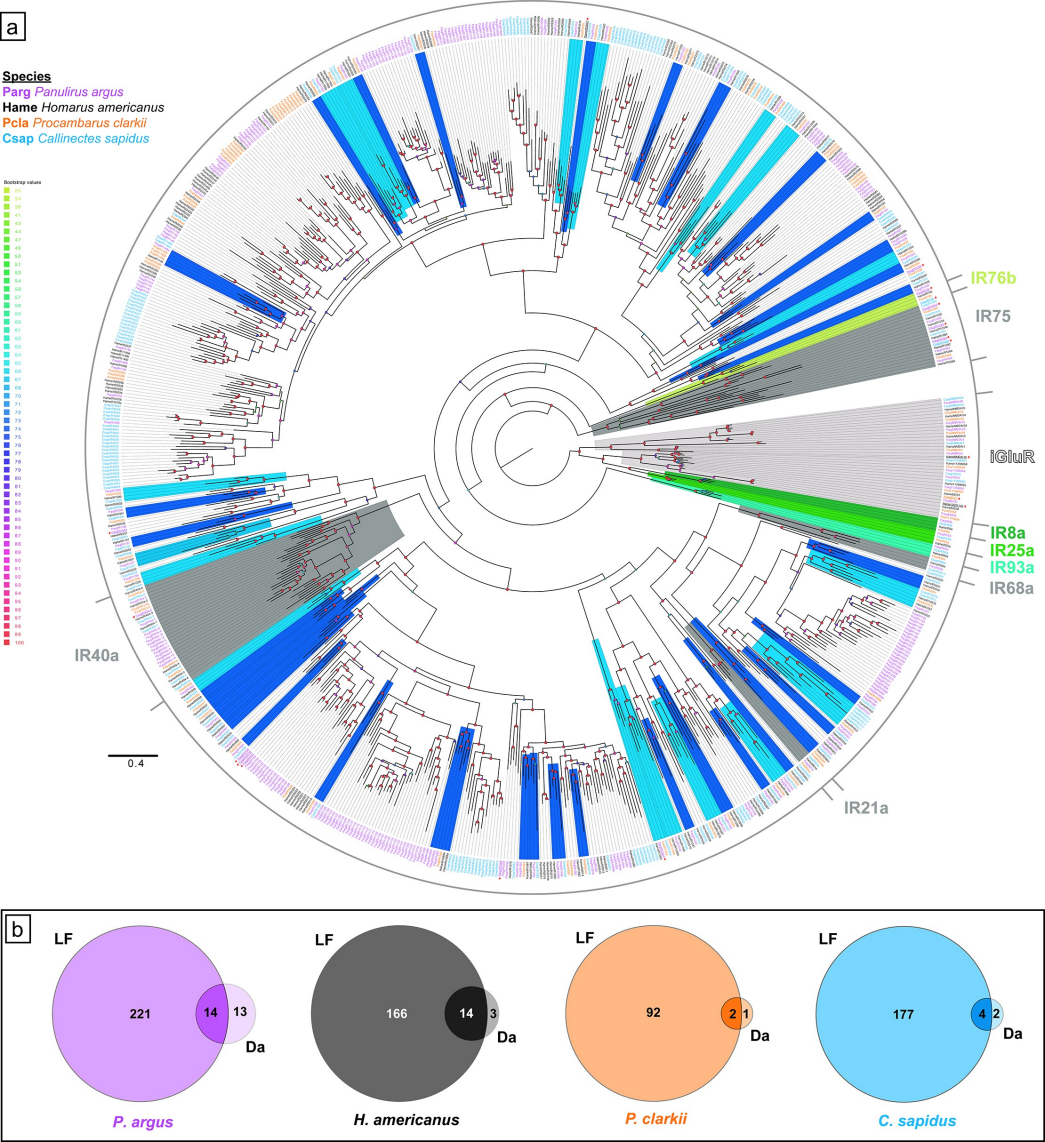
Phylogenetic tree of IRs in four decapod species and tissue expression. **a.** Maximum likelihood phylogenetic tree of IRs and iGluRs from four decapod crustaceans. Clades with co-receptor IRs (IR25a, IR8a, IR76b, and IR93a) are colored in shades of green; clades with tuning IRs that are conserved across crustaceans and insects (IR21a, IR40a, IR68a, and IR75-family) are colored dark grey; clades with tuning IRs that are conserved across all four decapod crustaceans are colored light blue; clades with tuning IRs that are conserved in at least three decapod crustaceans are colored dark blue; clades with iGluRs are colored light grey. * with underline indicates higher expression in dactyl than LF. The tree was built using IQ-Tree with 1000 UFBoot replications under the WAG+F+G4 model of substitution according to BIC as selected by ModelFinder. The tree was visualized on FigTree v.1.4.4. The tree is unrooted but the root is drawn at the iGluR/IR25a/IR8a clade. Scale bar represents expected number of substitutions per site. **b.** Venn diagrams showing tissue specific differential expression (**LF**–lateral flagella of antennules, **Da**–dactyls of walking legs) of IRs and iGluRs in each decapod crustacean as calculated by DESeq2, where a ~ 2.8 fold difference or greater in expression (i.e. log_2_[fold change] ≥ 1.5 or log_2_[fold change] ≤ -1.5) between tissue types is considered higher expression in one tissue.

**Fig 5:**
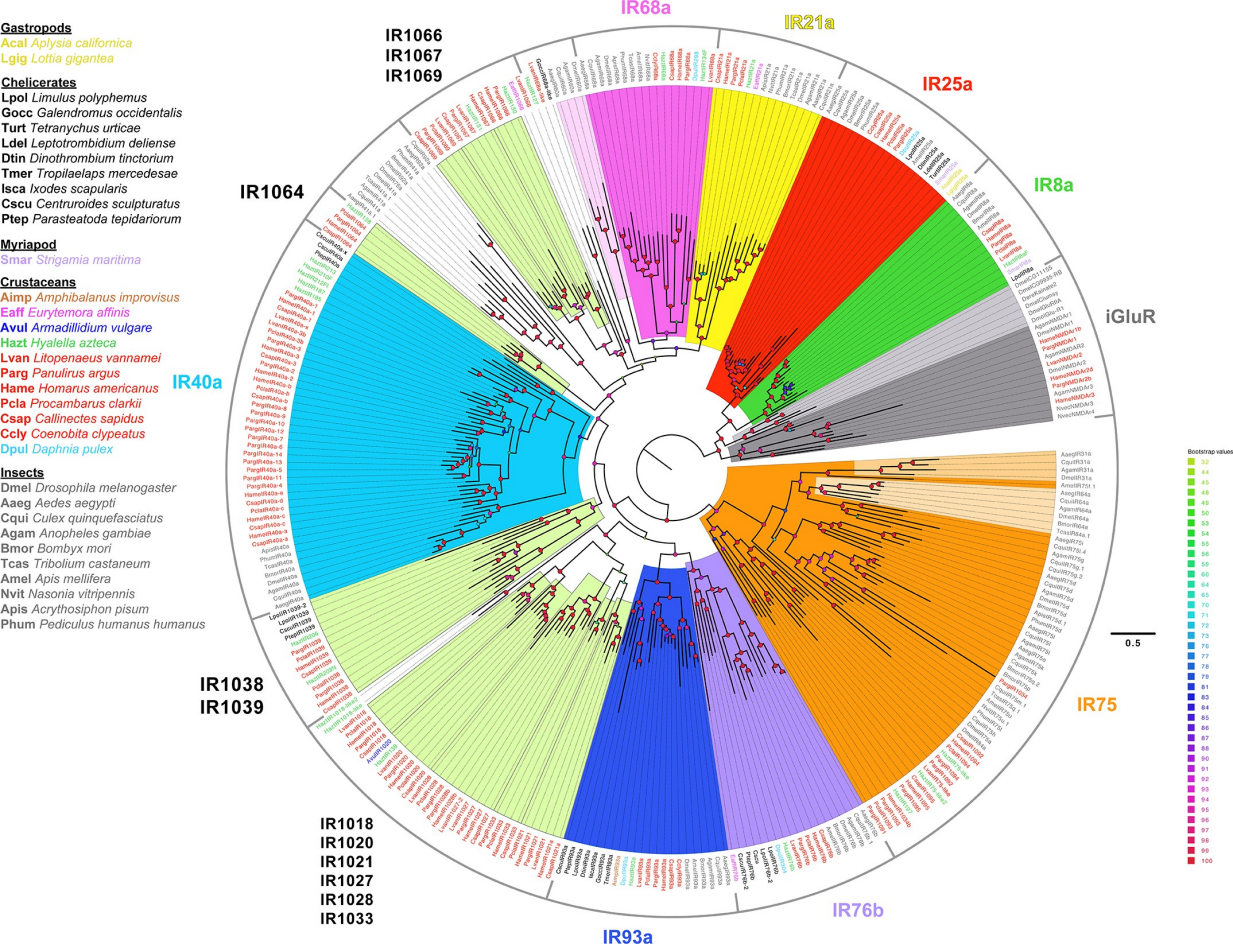
Phylogenetic tree of conserved IRs across arthropods. Maximum likelihood phylogenetic tree shows the different IRs that are conserved across major groups of arthropods: chelicerates, myriapods, crustaceans, and insects. IR25a sequences from two gastropods are also included. Among crustaceans, species are colored by their subclass as follows: thecostracan–brown; copepods–pink; isopods–navy blue; amphipods–green; decapods–red; branchiopod–fluorescent blue. The tree was built using IQ-Tree with 1000 UFBoot replications under the LG+F+G4 model of substitution according to BIC as selected by ModelFinder. The tree was visualized on FigTree v.1.4.4. The tree is unrooted but the root is drawn at the iGluR/IR25a/IR8a clade. Scale bar represents expected number of substitutions per site.

We performed phylogenetic analyses of sequences with both domains of iGluRs and IRs (referred as ‘selected sequences’) from the four decapod crustaceans (Fig 4; S10–S12 Files) and other arthropods (Fig 5; S13–S15 Files) to better determine iGluR and IR homologues. Table 2 summarizes these findings.

**Table 2:** Number of predicted IRs and iGluRs in transcriptomes from four species of decapod crustaceans.

Species	iGluRs	IRs			
		Co-receptor	Tuning IRs		
			Conserved	Species-Specific	Total
***Panulirus argus***	10	4	85	169	254
***Homarus americanus***	11	4	128	53	181
***Procambarus clarkii***	9	4	73	19	92
Callinectes sapidus*	6	4	87	99	186

Conservative estimate of number of IRs and iGluRs expressed in the transcriptomes of the four decapod crustaceans. iGluRs include NMDA and non-NMDA receptor sequences. Co-receptor IRs are IR25a, IR8a, IR76b, and IR93a. Conserved tuning IRs are variant tuning IR sequences that have homologues in other species. Most sequences accounted here have both domain regions representative of iGluRs and IRs. Some incomplete sequences that are homologous to conserved IRs in another species are also included (S10–S12 Files).

* indicates transcriptome generated from LF and dactyls only, while transcriptomes from the other species also include reads from brain tissue.

The phylogenetic tree of IRs and iGluRs in decapods is shown in Fig 4 and S1 Fig in polar tree and radial tree configurations, respectively. IRs and iGluRs in decapods are distributed into nine broad clades (S1 Fig). One clade has iGluRs, co-receptor IRs, IR75-family (IR1091–IR1095 and IR1034), IR1035, and IR1036. The IR40a family forms its own clade. The other seven clades are all tuning IRs only. Most of these clades have conserved tuning IRs, which are described below.

### iGluRs

The four decapod species expressed a number of iGluRs in their transcriptomes, ranging from six in *C*. *sapidus* to eleven in *H*. *americanus*. These iGluRs included NMDA receptor homologues NMDAr1, NMDAr2, and NMDAr3, and non-NMDA receptor homologues (Table 2). The identity and distribution of these iGluRs are shown in S3 Table, and their phylogenetic relationships are shown in Fig 4.

### Co-receptor IRs

The four co-receptor IRs–IR25a, IR8a, IR93a, and IR76b –were found in both chemosensory organs of all four decapod crustacean species (Fig 4, Table 2). According to RSEM analysis (S4–S7 Tables), IR25a is the most abundantly expressed IR in all four species.

DESeq2 analysis was used to identify quantitative differences in expression of these four co-receptor IR transcripts between tissue types for a given species. A difference in expression of transcripts between tissue types that is greater than ~ 2.8 fold (i.e., log_2_ [fold change] that is ≥ 1.5 or ≤ -1.5) is considered to be higher expression in a particular tissue type. The results of this analysis (S8–S11 Tables) show that IR25a is more highly expressed in LF than dactyl in all four species: in *P*. *argus*, 16 fold difference; in *H*. *americanus*, 356 fold difference; in *P*. *clarkii*, 121 fold difference, and in *C*. *sapidus*, 103 fold difference.

Like IR25a, IR93a is more abundantly expressed in LF than dactyl in all four species: in *P*. *argus*, 385 fold difference; in *H*. *americanus*, 1323 fold difference; in *P*. *clarkii*, 154 fold difference; and in *C*. sapidus, 4211 fold difference.

IR8a is more highly expressed in LF than dactyl in two of the species–*P*. *clarkii* and *C*. *sapidus*: in *P*. *clarkii*, 9.3 fold difference; and in *C*. *sapidus*, 5.8 fold difference. But in *P*. *argus* and *H*. *americanus*, there is no difference in expression in LF and dactyl.

IR76b has a more varied expression pattern between the tissue types in each species. In *P*. *clarkii*, IR76b has greater expression in LF than in dactyl by 34 fold. In *H*. *americanus*, there is no difference in expression of IR76b between the tissues. In *P*. *argus* and *C*. *sapidus*, IR76b has greater expression in dactyl than LF: 23 fold in *P*. *argus* and 3.0 fold in *C*. *sapidus*.

### Tuning IRs

Each species expresses many tuning IRs in the LF and dactyl: *P*. *argus* has 254 tuning IRs, *H*. *americanus* has 181, *P*. *clarkii* has 92, and *C*. *sapidus* has 186 (Table 2). It is important to note that these conservative estimates are based only on the number of sequences found in the transcriptome of each species that had both domain regions (PF00060 and PF10613) that define a variant IR. Some incomplete sequences were also included in this analysis due to homology to IRs with both domain regions from another species. If all the sequences that have only one of these domain regions are also taken into account, then the number of tuning IRs in each species almost doubles or more (Table 1).

Under the assumption that genes have not been missed in our transcriptome sequencing and assembly, some tuning IRs appear to be conserved phylogenetically, while some are unique to one of the four decapod species (Figs 4 and 5, Table 2). Therefore, there exist sub-classes of ‘conserved tuning IRs.’ Seventeen IRs have homologues in all four species; these are IR1001, IR1018, IR1020, IR1021, IR1029, IR1033, IR1037, IR1038, IR1039, IR1044, IR1046, IR1053, IR1057, IR1064, IR1065, IR1097, and IR1155. Out of these seventeen IRs, five IRs also have homologues in the decapod crustacean, *Litopenaeus vannamei* (Pacific white shrimp), three also have homologues in the amphipod, *Hyalella azteca*, one (IR1067) has homologues in both *L*. *vannamei* and *H*. *azteca*, and one (IR1020) has homologues in *L*. *vannamei*, *H*. *azteca*, and *Armadillidium vulgare* (Fig 5). Thus, we consider eleven IRs as “decapod-conserved” tuning IRs (IR1001, IR1018, IR1021, IR1029, IR1037, IR1044, IR1046, IR1053, IR1057, IR1097, IR1155) and five IRs as “crustacean-conserved” tuning IRs (IR1020, IR1038, IR1064, IR1066, IR1067) (Fig 5). Another IR, IR1069, detected in three species (*P*. *argus*, *P*. *clarkii*, and *C*. *sapidus*) also has a homologous sequence in *L*. *vannamei* and is therefore considered “decapod-conserved.” Four tuning IRs families that are conserved in insects–IR21a, IR40a, IR68a, and IR75 –were previously identified in *P*. *argus* [11]. While *P*. *clarkii*, *H*. *americanus*, and *C*. *sapidus* have homologues to IR21a, IR40a, and IR75-family, only *H*. *americanus* and *C*. *sapidus* also have homologues to IR68a (Figs 4 and 5). Similar to *P*. *argus* [11], *P*. *clarkii*, *H*. *americanus*, and *C*. *sapidus* have an expanded family of IR40a homologues (Figs 4 and 5). In all four decapods, IR21a is more abundantly expressed in LF compared to dactyl, and all IR40a homologues are more abundantly expressed in LF compared to dactyl with three exceptions: IR40a-3 in *P*. *argus* is more highly expressed in dactyl compared to LF; IR40a-8 in *P*. *argus* and IR40a-e in *H*. *americanus* have similar expression in both LF and dactyl (S4–S11 Tables). IR68a homologues are more abundantly expressed in LF compared to dactyl in all three species that express it (*P*. *argus*, *H*. *americanus*, and *C*. *sapidus*). We consider sequences that are numbered IR1091–IR1095 and IR1034 in the decapod crustaceans to be homologous to the insect IR75-family of genes. For the IR75-family, in *P*. *argus*, IR1091–IR1093 and IR1034 have higher expression in dactyl compared to LF, and IR1094 and IR1095 have similar expression in LF and dactyl. In *H*. *americanus*, IR1034, IR1092, and IR1095 have higher expression in LF compared to dactyl, and IR1034b, IR1091, and IR1094 have similar expression in LF and dactyl. In *P*. *clarkii*, both IR1093 and IR1094 have higher expression in LF compared to dactyl. In *C*. *sapidus*, IR1091, IR1093, and IR1095 have higher expression in LF compared to dactyl, while IR1092 has higher expression in dactyl than LF (S4–S11 Tables). We also found homologues to these four conserved IRs (IR21a, IR40a, IR68a, and IR75-family) in *L*. *vannamei* and *H*. *azteca*.

Among chelicerates, we found homologues to IR25a, IR76b, and IR93a in several species (Fig 5). However, we found IR8a only in *Limulus polyphemus*, similar to Vizueta et al. (2018) [12]. We also identified putative homologues to IR40a in *Parasteatoda tepidariorum* and *Centruroides sculpturatus*, and putative homologues to IR68a in *Galendromus occidentalis*. We identified putative homologues to the tuning IR, IR1039, which has homologues in all four decapod crustaceans and in *H*. *azteca*, *L*. *polyphemus*, *P*. *tepidariorum*, and *C*. *sculpturatus* (Fig 5). As IR21a, IR40a, IR68a, and IR75-family are found in at least two major groups of arthropods–Crustacea and Insecta (Pancrustacea), we consider these tuning IRs as “arthropod-conserved.” Similarly, IR1039 is also considered an “arthropod-conserved,” since it is found in crustaceans and chelicerates. The remaining tuning IRs from the four decapod crustaceans, which include most of them, were expressed in one to four of the decapod species examined in our study. Thirty-four IRs have homologues in at least three decapod species, and many IRs have homologues in at least two species (Fig 4, Table 2). IRs with homologues in all four decapod species might be a conservative estimate of “decapod-conserved” IRs while IRs with homologues in two of the four decapods is a more liberal estimate of “decapod-conserved” IRs. Species-specific IRs are operationally defined as those expressed in only one of the four decapod species and without homologues being found in other arthropod species (Fig 4, Table 2).

The DESeq2 analysis showed that for all four species, most of the tuning IRs are more highly expressed (> ~ 2.8 fold difference) in one or the other chemosensory organ (Fig 4B), and almost without exception, they were more highly expressed in the LF than dactyl (S4–S11 Tables). None of the dactyl-enriched tuning IRs (Fig 4) have homologues in all four species. The distribution and expression of IRs across the tissue types and species are diverse. The only consistent pattern among the four decapod species is that almost all IRs found so far are more highly expressed in the LF than dactyl (S4–S11 Tables).

Some IRs previously identified in various insect species (e.g. IR31a, IR60a, and IR64a) [3, 19] were searched for but not identified in these decapod transcriptomes, and thus they are probably “insect-conserved” tuning IRs.

### Immunolocalization of IR25a in LF and dactyl of *P*. *argus* and *H*. *americanus*

**Fig 6:**
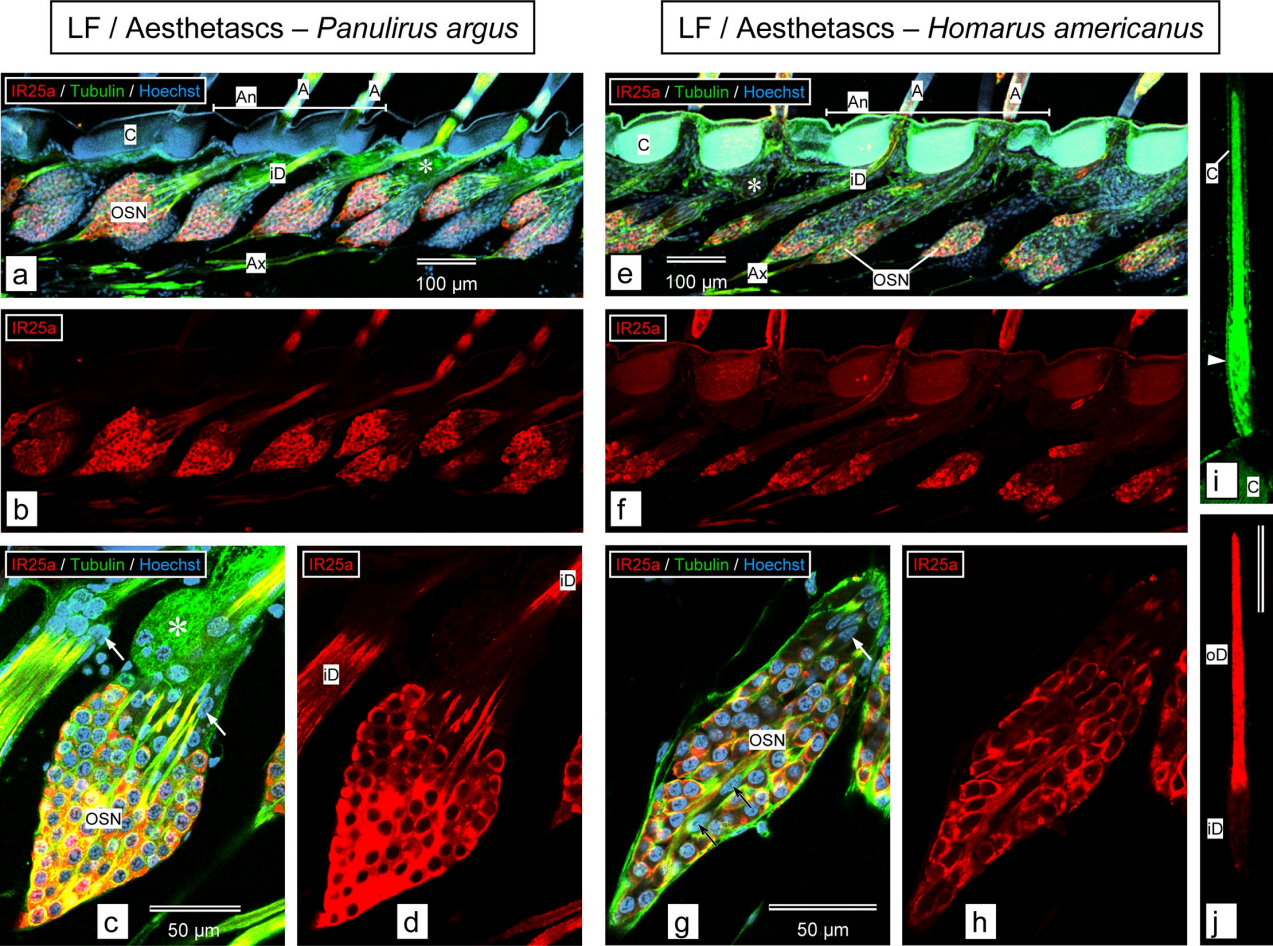
Immunolabeling with anti-HaIR25a in the aesthetasc-bearing tuft region of the lateral flagellum of the antennule. **a–d.** *Panulirus argus*. **e–j.** *Homarus americanus*. **a, b, e, f.** Sagittal sections through the medial plane of the tuft region of lateral flagellum labeled with anti-HaIR25a (red), anti-tubulin (green), and Hoechst 33258 (blue) at low magnification (maximum intensity projections of confocal image stacks). Scale bar in **a** also applies to **b**, scale bar in **e** also applies to **f**. **a** and **b** adapted from Kozma et al. (2018) [11]. **a, e.** Overlay of all 3 confocal channels. **b, f.** anti-HaIR25a channel. Overall organization of aesthetascs is similar in both species: two rows of aesthetasc setae (A) arise from the autofluorescent (blue in *P*. *argus*, blue and green in *H*. *americanus*) cuticle (C) of an annulus (An + horizontal bar). Each aesthetasc seta is associated with a large cluster of olfactory sensory neuron (OSN) somata which are distinctly labeled by anti-HaIR25a and labeled with moderate intensity by anti-tubulin. Bundles of inner dendritic segments (iD) arising at the apical pole of the OSN clusters are labeled with moderate intensity by both antibodies. Bundles of axons (Ax) arising at the basal pole of OSN clusters are intensely labeled by anti-tubulin labeled with moderate intensity by anti-HaIR25a. Tegumental glands (asterisks) are located between bundles of inner dendritic segments. OSN clusters in *H*. *americanus* are more elongated than in *P*. *argus* and contain fewer OSNs. **c, d, g, h.** Sagittal section through OSN clusters labeled with anti-HaIR25a (red), anti-tubulin (green), and Hoechst 33258 (blue) at high magnification (confocal images–maximum intensity projection of confocal image stacks with a total thickness of about 1 μm). Scale bar in **c** also applies to **d**, scale bar in **g** also applies to **h**. **c, g.** Overlay of all 3 confocal channels. **d, h.** anti-HaIR25a channel. The somata (OSN) of all OSNs identified by having almost spherical nuclei are distinctly labeled by anti-HaIR25a. Note that the overall shape of OSNs is close to spherical in *P*. *argus* but more elliptical in *H*. *americanus*. Somata of auxiliary cells (identified by having flat, elongated nuclei—arrows) are not labeled by anti-HaIR25a. In *H*. *americanus*, auxiliary cells are not only present at the apical pole of the OSN cluster (white arrows) but also in its center (black arrows). **i, j.** Horizontal section through an aesthetasc of *H*. *americanus* labeled with anti-HaIR25a (red) and anti-tubulin (green) (confocal images of one optical section of 1 μm thickness). Scale bar in **j** (100 μm) also applies to **i**. Note that anti-tubulin non-specifically labeled cuticle (C) in addition to dendrites enclosed in the thin cuticular tube of the aesthetasc seta. The bulge at the bottom of the seta (arrowhead) indicates the transition region between inner dendritic segments (iD) and outer dendritic segments (oD). Note that labeling intensity of anti-HaIR25a is considerably higher in oD compared to iD.

We compared the expression of IR25a in sensory neurons in the LF and walking leg dactyls of *P*. *argus* and *H*. *americanus*. The general organization of aesthetascs and the cells associated with them is similar for *P*. *argus* (Fig 6A, [11]) and *H*. *americanus* (Fig 6E). Each aesthetasc is innervated by ca. 300 OSNs, whose somata form a cluster near the base of the seta, with dendrites extending into the sensilla and axons projecting to the brain. Besides OSNs, there are two other cell types associated with the aesthetascs, and these are easily distinguishable based on their position and shape [82]. Auxiliary cells ensheathe the bundle of OSN inner dendritic segments, forming a strand of flat lenticular nuclei arranged in a tube-like fashion around those dendrites (Fig 6C and 6G: arrows). A second cell type is the tegumental gland cells, which are located in the spaces between the OSN somata clusters and the dendritic bundles (Fig 6A, 6C and 6E: asterisks), and which form distinctive rosettes with a duct projecting from the gland to the cuticular surface.

For the LF, in *P*. *argus*, anti-HaIR25a intensely labeled the clusters of OSN somata associated with the aesthetascs (Fig 6A–6D). Simultaneous labeling with anti-tubulin (to preferentially label neurons–see Methods) and Hoechst 33258 (to label nuclei of all cells) revealed that all or close to all OSN somata of a cluster are HaIR25a-positive as long as they are not so deep in the cluster that the antibody cannot penetrate to react with them (see Methods) while adjacent auxiliary cells are HaIR25a-negative (Fig 6C and 6D). The HaIR25a-like immunoreactivity is present in the cytosol and cell membrane of the OSN somata, as well as in the inner- and outer dendritic segments (Fig 6D). In *H*. *americanus*, anti-HaIR25a also labeled the clusters of OSN somata associated with the aesthetascs of the LF, but with slightly lower intensity (Fig 6E–6H). Like in *P*. *argus*, simultaneous labeling with anti-tubulin and Hoechst 33258 revealed that all or close to all OSN somata of a cluster are HaIR25a-positive while auxiliary cells, some of which are located within the cluster of OSN somata, are HaIR25a-negative (Fig 6G and 6H). In the OSN somata, HaIR25a-like immunoreactivity is most uniform and intense in the cell membrane, whereas labeling of the cytosol is more variable in intensity (Fig 6H). As in *P*. *argus*, HaIR25a-like immunoreactivity extends into the dendrites of the OSNs where it is distinctly more intense in the outer- compared to the inner dendritic segment (Fig 6I and 6J).

**Fig 7:**
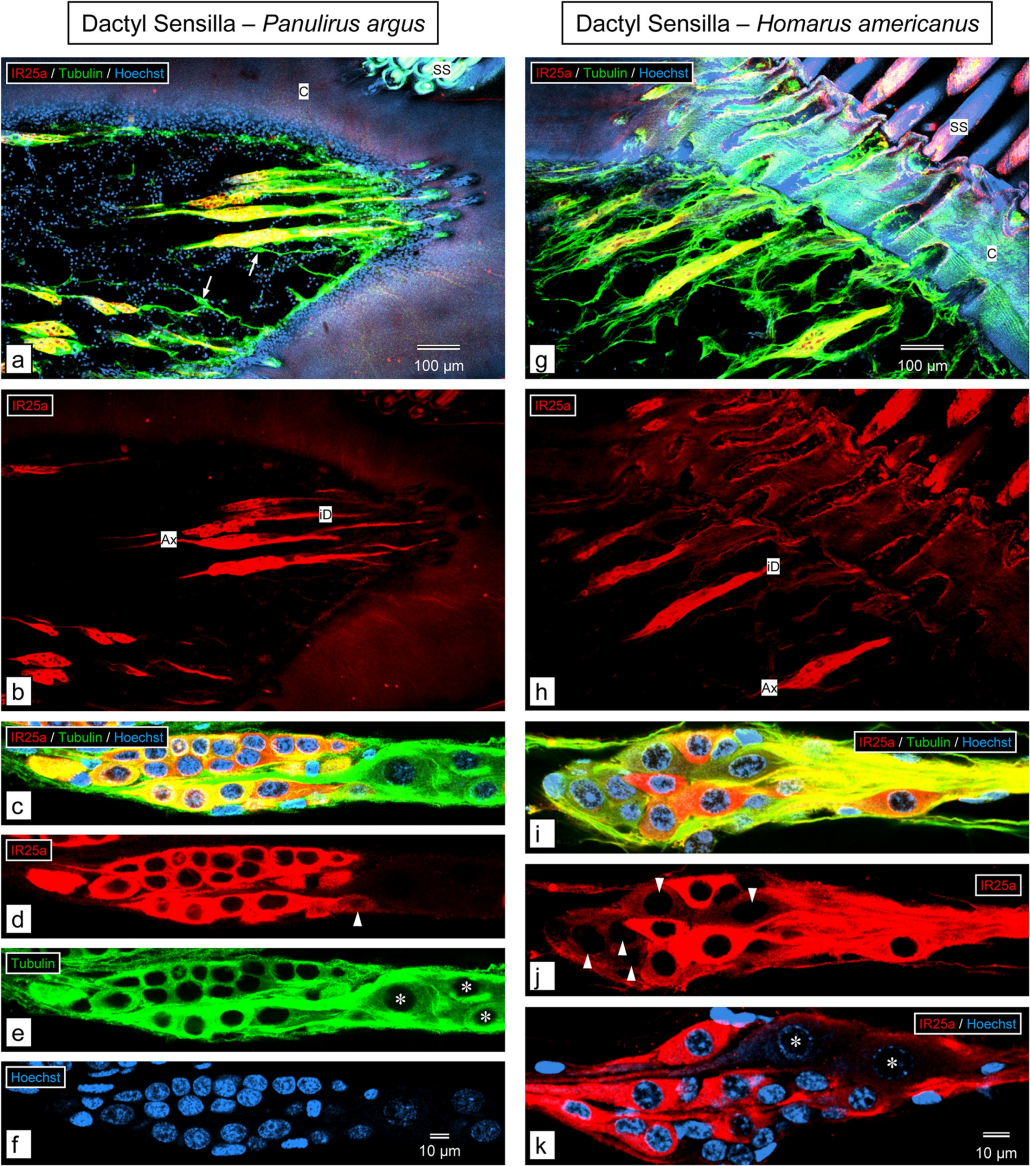
Immunolabeling with anti-HaIR25a in the walking leg dactyl. **a–f.** *Panulirus argus*. **g–k**. *Homarus americanus*. **a, b, g, h.** Sagittal sections through distal part (excluding the epicuticular cap) of the dactyl of the 3^rd^ pereiopod labeled with anti-HaIR25a (red), anti-tubulin (green), and Hoechst 33258 (blue) at low magnification (maximum intensity projections of confocal image stacks that are 5–10 μm thick). Scale bar in **a** also applies to **b**, scale bar in **g** also applies to **h**. **a, g.** Overlay of all 3 confocal channels. **b, h.** anti-HaIR25a channel. In both species, the numerically dominant sensilla are smooth setae (SS) organized into large, distinct groups. Each smooth seta is innervated by an elongated cluster of sensory neurons that is more than 200 μm long and about 50 μm in diameter and intensely labeled by anti-HaIR25a and anti-tubulin. Both antibodies label the somata of sensory neurons as well as their axons (Ax) and inner dendritic segments (iD). Note that in *P*. *argus*, but not in *H*. *americanus*, single bipolar sensory neurons labeled by anti-tubulin but not anti-HaIR25a (arrows) are interspersed between the double-labeled clusters of sensory neurons. **c–f, i—k.** Two examples of clusters of sensory neurons labeled with anti-HaIR25a (red), anti-tubulin (green), and Hoechst 33258 (blue) at high magnification (maximum intensity projections of two adjacent optical sections of 0.4 μm thickness); scale bar in **f** also applies to **c–e**; scale bar in **k** also applies to **i** and **j. c, i.** Overlay of all three channels. **d, j.** anti-HaIR25a channel. **e.** anti-tubulin channel. **f.** Hoechst channel. **k.** Overlay of anti-HaIR25a and Hoechst channel.

For the walking leg dactyls, in *P*. *argus*, anti-HaIR25a labeled the fusiform clusters of somata of sensory neurons associated with the clustered smooth setae (Fig 7A–7F). Simultaneous labeling with anti-tubulin and Hoechst 33258 revealed that almost all somata of putative CSNs (nuclei characterized by dense heterochromatin) in the proximal part of each sensory neuron cluster are intensely labeled by anti-HaIR25a, while the remaining 1–3 putative CSNs are less intensely labeled. In addition to the putative CSNs, each cluster of sensory neurons contains 2–3 larger somata of putative MSNs (nuclei characterized by very loose heterochromatin) at its distal pole that are HaIR25a-negative (Fig 7C–7F). In *H*. *americanus*, anti-HaIR25a also labeled the fusiform clusters of somata of sensory neurons associated with the tufts of smooth setae of the walking leg dactyls (Fig 7G–7K). Simultaneous labeling with anti-tubulin and Hoechst 33258 revealed that in contrast to the situation in *P*. *argus*, only about half of the somata of putative CSNs (nuclei characterized by dense heterochromatin) in the proximal part of each sensory neurons cluster are intensely labeled by anti-HaIR25a while the remaining half are only lightly labeled (Fig 7I and 7J). As in *P*. *argus*, each cluster of sensory neurons contains 2–3 larger somata of putative MSNs (nuclei characterized by very loose heterochromatin) at its distal pole that are HaIR25a-negative (Fig 7K).

In both species, each cluster contains about 20 bipolar sensory neurons. In *P*. *argus*, all sensory neurons are strongly labeled by anti-tubulin, but in *H*. *americanus*, the labeling with anti-tubulin is not as intense and more diffuse. In *P*. *argus*, all sensory neurons located in the proximal part of the cluster are also intensely labeled by anti-HaIR25a, but three particularly large neurons located at the distal pole of the cluster are not labeled by anti-HaIR25a (nuclei labeled by asterisks), and one additional neuron in the distal region is only weakly labeled by anti-HaIR25a (arrowhead). In contrast, in *H*. *americanus*, only some of the sensory neurons in the proximal part of the cluster are intensely labeled by anti-HaIR25a and about equally many are weakly labeled (arrowheads). Two particularly large neurons located at the distal pole of the cluster are not labeled by anti-HaIR25a (nuclei labeled by asterisks). Note that in both species, the nuclei of the large HaIR25a-negative neurons at the distal pole of the cluster (asterisks) are characterized by very loose heterochromatin, whereas the heterochromatin of the other sensory neurons is considerably denser.

### TRP channels

**Fig 8:**
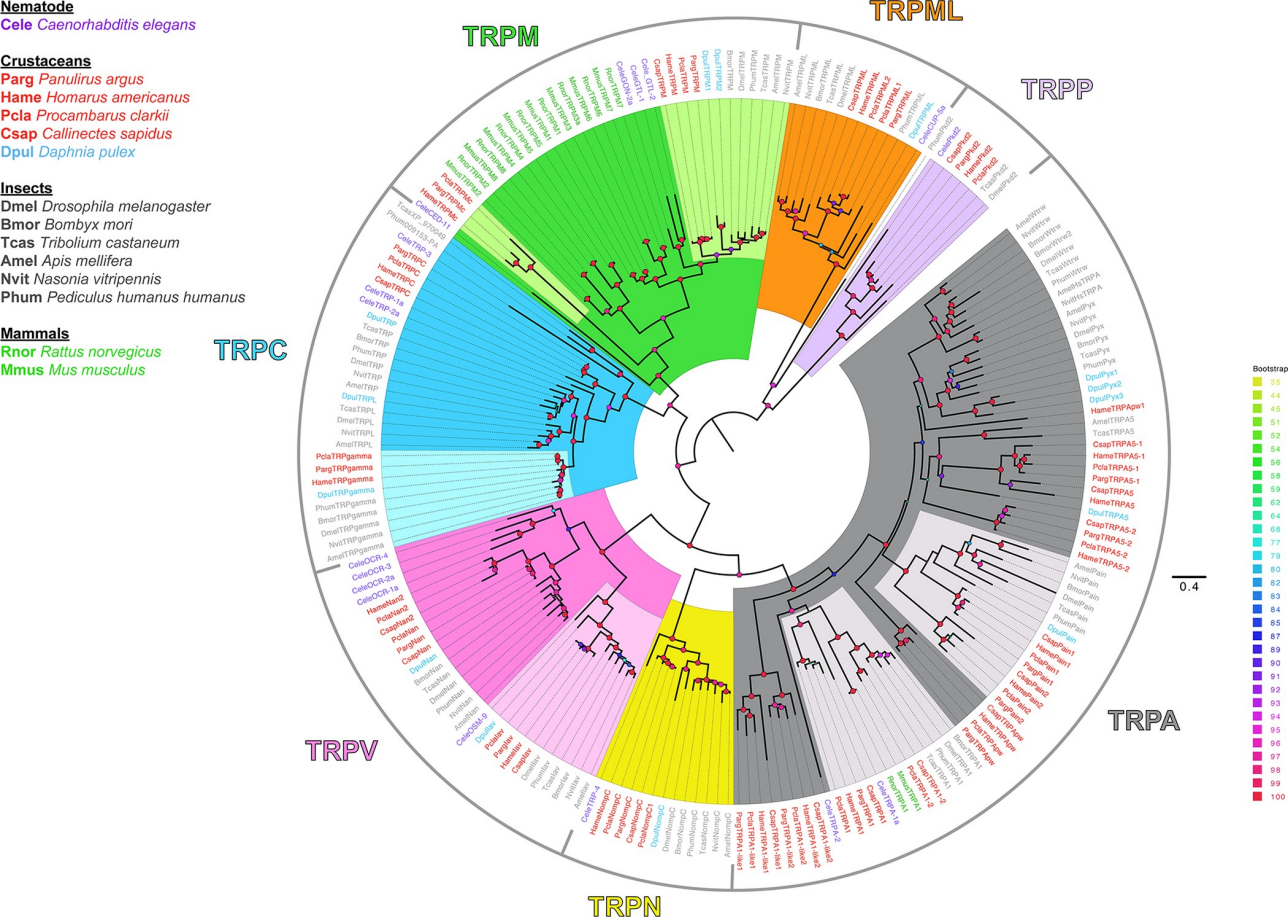
Phylogenetic tree of TRP channels across animals. The maximum likelihood phylogenetic tree shows the conservation of TRP channel sequences from the transcriptomes of four decapod crustaceans with TRP channels from insects, nematodes, and mammals. Among crustaceans, decapods are in red and branchiopod in light blue. All four decapod crustaceans have several homologues to each subfamily of TRP channels. The tree was constructed on IQ-Tree with 1000 UFBoot replications under the LG+F+G4 model of substitution according to BIC, as determined by ModelFinder. Tree was visualized on FigTree v.1.4.4. Tree was unrooted but is drawn with the Group 2 subfamilies, TRPML and TRPP, clades as the root. Support for some inner nodes is low due to incomplete sequences and high divergence. Scale bar represents expected number of substitutions per site.

Homologues to all seven subfamilies in both groups of TRP channels (see Fig 1) were previously found in the LF and dactyl transcriptomes for *P*. *argus* [11]. Homologues of all seven subfamilies were also found in the three additional crustacean transcriptomes analyzed here (Fig 8, S16–S18 Files). Within each subfamily of TRP channels, those from decapod crustaceans form their own cluster to corresponding homologous clusters of TRP channels from other species. In Group 1 TRP channels of arthropods, the TRPA subfamily has an expanded family of channels, and a similar expansion was found in these decapod crustaceans. Similar to insects, the TRPA family in the four decapods is expanded with homologues to TRPA1, painless, TRPA5, and pyrexia/waterwitch. *H*. *americanus* has one additional sequence (HameTRPApw-1) that is more closely related to the pyrexia/waterwitch clade, but with low bootstrap support, which was not detected in the other decapod crustaceans. Additionally, the four decapod crustaceans also have TRPA sequences that do not have homologues in insects (TRPA1-like1 and TRPA1-like2). Unlike insects, there are two genes that belong to the TRPM subfamily in crustaceans. Homologues to the insect TPRM channel were found in all four species. *P*. *argus*, *H*. *americanus*, and *P*. *clarkii* have an additional TRPM channel (TRPMc–previously denoted as TRPMm in Kozma et al. 2018 [11]) (Fig 8), which clusters away from the arthropod conserved group of TRPM channel sequences, while *C*. *sapidus* has only the insect-like TRPM channel. The branchiopod *Daphnia pulex* has two TRPM channels as well; however, both are more closely associated with the insect TRPM channel. Therefore, it is possible that the TRPMc channel is specific to decapods, but this needs to be resolved by the inclusion of TRPM channels from other crustaceans and protostomes in analyses.

Among the TRPC subfamily, there were two homologues in the decapods. Homologues to TRPgamma were detected in *H*. *americanus* and *P*. *clarkii* along with the homologue previously discovered in *P*. *argus*. Homologues to the TRPC channel previously detected in *P*. *argus* were also detected in the other three decapods. In the TRPN subfamily, homologues to NompC were detected in all four species, along with an additional TRPN channel that was found only in *P*. *clarkii*. In the TRPV subfamily, homologues to Inactive (Iav) and Nanchung (Nan) were found in all four species. An additional Nanchung homologue was also detected in *P*. *clarkii* and *C*. *sapidus*.

Homologues of Group 2 TRP channels–TRPP (Pkd2) and TRPML–were also found in all four decapod species. Two TRPML sequences were detected in *P*. *clarkii*: TRPML1 and TRPML2.

Based on DESeseq2 analysis, in *P*. *argus*, TRPA5-1 and TRPgamma have higher expression in LF compared to dactyl, while NompC, Iav, and Nan have higher expression in dactyl compared to LF. In *H*. *americanus*, NompC, TRPgamma, and TRPC have higher expression in LF compared to dactyl, while TRPApw and Iav have higher expression in dactyl than LF. In *P*. *clarkii*, almost all TRP channels have differential expression between the LF and dactyl tissues. TRPML1, TRPML2, NompC, and NompC1 have higher expression in dactyl than LF, while TRPA5-1, TRPA5-2, Pain2, TRPA-like, TRPA1, Iav, Nan, Nan2, TRPC, and TRPMc have higher expression in LF compared to dactyl. In *C*. *sapidus*, Nan2 has higher expression in dactyl than LF, while TRPA1-like, TRPA1-like1, and TRPC have higher expression in LF compared to dactyl. The remaining TRP channels in all four decapod species studied here have similar expression in LF and dactyl.

### GRs and GRLs

**Fig 9:**
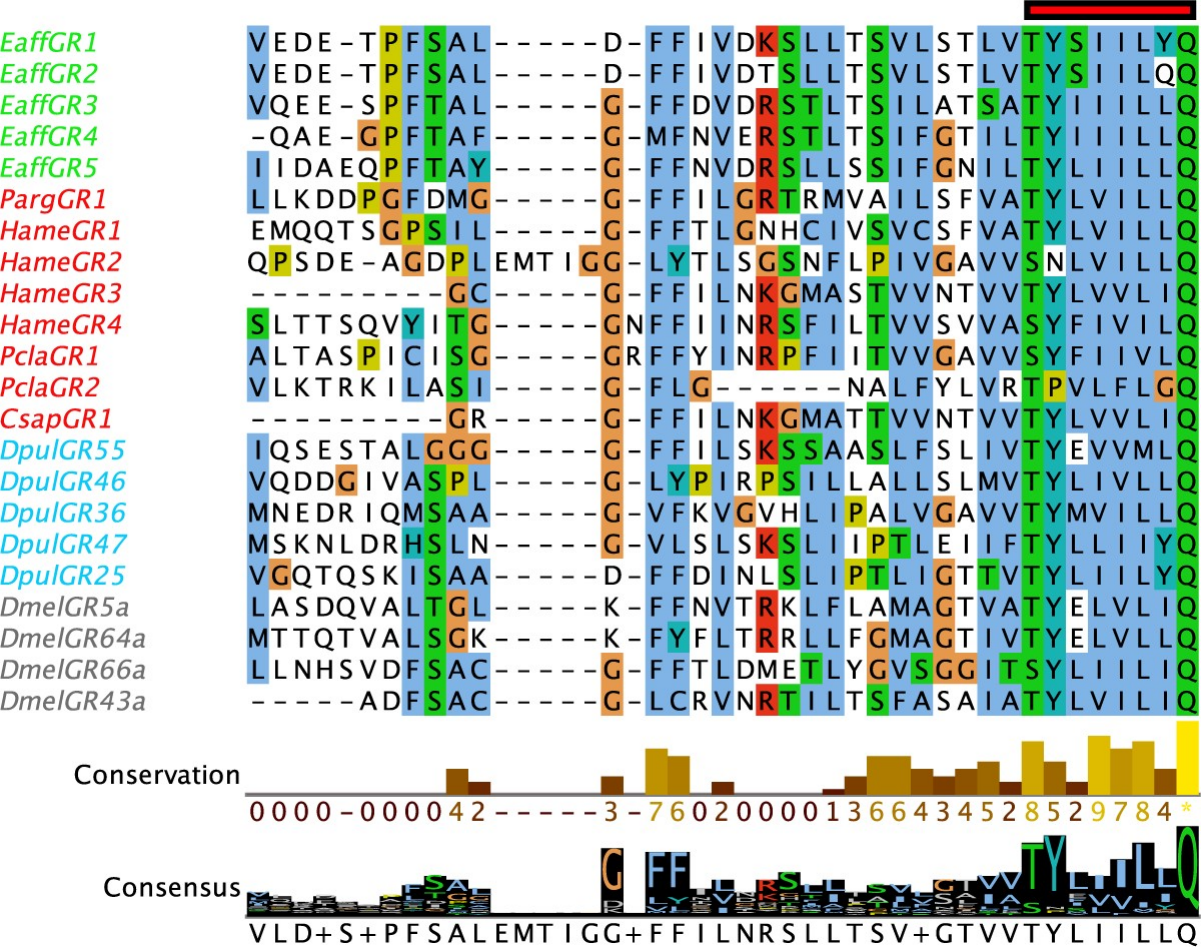
Multiple sequence alignment of GRL fragments in decapod crustaceans and GRs in arthropods. Multiple sequence alignment shows the TM7 region of the sequences that have the highly conserved “TYxxxxxQF” motif (red bar). Sequences were aligned using MAFFT and visualized on Jalview. Decapod crustaceans are in red. Green–Eaff–*E*. *affinis*; Blue–Dpul–*D*. *pulex*; Grey–Dmel–*D*. *melanogaster*. Conservation of amino acids and the consensus histogram were annotated on Jalview. Clustal X color scheme was used to color residues.

Using InterProScan for domain search of the 7tm_7 domain region (PF08395) and the criteria for identification of GRs and GRLs as described by Robertson [6], we detected GRLs in each of the four decapod species (Fig 9, S19 and S20 Files). The number of GRLs range from one to four across the four decapods. We did not detect homologues to these GRLs in other crustaceans or insects. There is one GRL, PargGR1, identified previously in *P*. *argus* [11]. Although PargGR1 has low expression in the transcriptome, it is three fold more abundant in the LF than dactyl (S4 and S8 Tables). In *H*. *americanus*, there are four GRLs: HameGR1 is expressed in both tissues and is 10 fold more abundant in dactyl than LF; HameGR2 has similar expression in both tissues; HameGR3 has low expression in LF; and HameGR4 expression is low but similar in both tissues. In *P*. *clarkii*, there are two GRLs: PclaGR1 has low level of expression in both tissues without much difference in abundance; and PclaGR2 has a low level of expression and only in the dactyl. *C*. *sapidus* has only one GRL, CsapGR1. CsapGR1 was identified through InterProScan in the transcriptome before redundancy was removed; however, it was not subsequently detected in the non-redundant transcriptome.

### Expression of putative chemoreceptor proteins in the brain

We previously discovered expression of several IRs including IR25a, IR8a, and IR93a in the brain transcriptome of *P*. *argus* and found evidence of expression of these co-receptor IRs in the brain through PCR [11]. Several other conserved IRs and tuning IRs with low expression were also detected in the brain transcriptome. Using immunocytochemistry, we discovered that IR25a is only localized to very large and conspicuous cells in the axon sorting zone in the lateral division of the antennular nerve entering the brain in *P*. *argus* [11]. Here, we generated brain transcriptomes and detected expression of all four co-receptor IRs in *H*. *americanus* and *P*. *clarkii*, similar to *P*. *argus*. We also detected expression of IR21a, IR40a-family, and IR75-family in the brains of these three species. In *H*. *americanus*, we also detected expression of IR68a. Twenty to fifty tuning IRs are expressed in the brain transcriptomes of *P*. *argus*, *H*. *americanus*, and *P*. *clarkii*, with *P*. *clarkii* on the lower end of the range and *H*. *americanus* on the higher. In *P*. *argus*, there are no IRs that have higher expression in the brain compared to LF with the exception of IR1093 from the IR75-family: expression of IR1093 in the brain is 28 fold higher than LF and 4.9 fold higher than dactyl. In *H*. *americanus*, IR2067 has 21 fold higher expression in brain than LF, IR2032 in the brain has 4.3 fold higher expression than LF and 3.1 fold higher expression than dactyl, IR2005 in the brain has 7.9 fold higher expression than dactyl, and IR1034, IR1092, and IR1095 of the IR75-family have 8.6 fold, 6.1 fold, and 8.7 fold higher expression respectively in the brain compared to dactyl. In *P*. *clarkii*, IR1094 of the IR75-family has 94 fold higher expression in the brain than dactyl.

All TRP channels from all subfamilies that were detected in the LF and dactyl of *P*. *argus*, *H*. *americanus*, and *P*. *clarkii* were also detected in their brain transcriptomes with varying levels of expression (S4–S6 Tables, S1 File). The only exception was the TRPN channel of *H*. *americanus*, NompC, which had little to no expression in the brain. In *H*. *americanus*, TRPgamma had higher expression in brain than LF and dactyl by 935 fold and 5000 fold respectively, and TRPC was 32 fold more abundant in brain than dactyl. In *P*. *clarkii*, TRPML1 and TRPML2 were 3.5 fold and 3.0 fold more abundant in brain than LF, Pain2 was more abundant in brain than dactyl by 8.6 fold, TRPA1-like2 in brain was more highly expressed than LF and dactyl by 5.3 fold and 6.2 fold respectively, TRPV channel Nan was expressed more highly in the brain than dactyl by 3.9 fold, TRPgamma was more abundantly expressed in brain than LF and dactyl by 352 fold and 358 fold respectively, and TRPC was more highly expressed in the brain than dactyl by 23 fold.

GR expression was found in the brain of *H*. *americanus* for HameGR1, HameGR3, and HameGR4. In fact, HameGR4 was much more abundantly expressed in the brain than in the LF or dactyl by 74 fold and 36 fold respectively.

## Discussion

Animals have a diversity of types of chemoreceptor proteins with expression patterns that differ phylogenetically. Arthropods are a major animal phylum, for which its largest clade–the insects–has been the focus of research on the molecular identity of chemoreceptors [8]. The other major clades of arthropods, including crustaceans, have received much less attention. Our work contributes to our understanding of the evolution of chemoreceptor proteins and helps support future research on crustacean chemoreception by a comparative analysis of chemoreceptor proteins in four species of decapod crustaceans that are used as models of chemoreception: Caribbean spiny lobster *Panulirus argus*, American lobster *Homarus americanus*, red swamp crayfish *Procambarus clarkii*, and blue crab *Callinectes sapidus*.

### Evolution and function of crustacean IRs

IRs are an ancient group of chemoreceptor proteins, being present in all protostomes and well represented in the decapod crustaceans. A conservative estimate of the number of different IRs expressed in the two chemosensory organs of the four species examined in our study, based on sequences that have both of the major domains of IRs, is ca. 250 for *P*. *argus*, ca. 170 *for H*. *americanus*, ca. 100 for *P*. *clarkii*), and ca. 180 for *C*. *sapidus*. Since IRs, like the iGluRs from which they evolved, are heterotetramers, these IRs exist in combinations to form functional channels. One or two of the constituent subunits are co-receptor IRs and the other two to three subunits are tuning IRs [16].

Homologues to four co-receptor IRs–IR25a, IR8a, IR76b, and IR93a –exist in the transcriptomes of both chemosensory organs of the four species of decapod crustaceans examined here. IR25a is the most ancient of IRs, being a protostome conserved IR that is absent from the deuterostomes and clades ancestral to the protostome-deuterostome split (i.e., Placozoa, Porifera, Cnidaria, and Ctenophora: [10, 18, 19]). IR8a is an arthropod conserved co-receptor IR. There is limited information about the expression of IR25a and IR8a in the crustaceans, unlike in the insects, for which OSNs are known to express either IR25a or IR8a, and sometimes both. IR93a is expressed only in the antenna of *Drosophila*, whereas IR76b is found in all of *Drosophila*’s chemosensory tissues [15]. More specifically, IR93a is co-expressed in 10 to 15 neurons surrounding the sacculus on the antenna of the fly. The tuning IRs IR40a, IR68a, and IR21a are co-expressed with IR93a in these neurons, and this combination of IRs specifies hygro- and thermosensation rather than chemoreception [20–23]. In crustaceans, studies of cellular expression using immunocytochemistry and/or *in situ* hybridization show that IR25a is broadly expressed in OSNs, being present in many or most OSNs in *P*. *argus*, *H*. *americanus*, and *C*. *clypeatus* [11, 31–34, 36]. IR25a is also broadly expressed in CSNs of various chemosensory organs of *P*. *argus* [11]. Our immunocytochemical results on *P*. *argus* show that IR25a is expressed in all or most OSNs and chemosensory neurons (CSNs) in the dactyls but not in mechanosensory neurons (MSNs). Furthermore, the labeling is strongest in the outer dendrites of the OSNs, where receptor proteins are highly expressed [85, 86]. Our results on *H*. *americanus* are largely similar, except that only about half of the dactyl CSNs are labeled. The reason for this difference in extent of expression of IR25a in dactyl CSNs between *P*. *argus* and *H*. *americanus* is not clear. Considering IR8a, its cellular expression pattern has not been described, though IR8a is known through PCR experiments to be expressed in several chemosensory and non-chemosensory organs in *P*. *argus* [11]. Furthermore, organ-level expression levels for IR8a appear to be lower than for IR25a, suggesting that IR8a is expressed in a more limited number of cells than is IR25a. IR93a is reported to be expressed in all or most OSNs of *P*. *argus*, based on in situ hybridization studies [34]. In insects, IR25a and IR8a are essential for targeting IRs to the OSN dendritic membrane and thus becoming a functional channel, and are not necessary for expression of the channels’ chemical specificity [16, 17]. It might be expected that crustacean co-receptor IRs act similarly, but that has not been studied.

The tuning IRs make up the vast majority of the IR repertoire in crustaceans, similar to insects. Conservatively, this is at least 254 in *P*. *argus*, 181 in *H*. *americanus*, 92 in *P*. *clarkii*, and 186 in *C*. *sapidus*. *Daphnia pulex* has ca. 150 tuning IRs, *Hyalella azteca* has 114, and *Eurytempora affinis* has 18 [3, 18, 19]. Some tuning IRs in crustaceans are more phylogenetically conserved than others. For example, to date, IR21a, IR40a, and IR75 have been found to have homologues only in pancrustaceans and thus are likely expressed only in crustaceans and insects. IR1039 is conserved in crustaceans and chelicerates, but is not detected in insects. Many tuning IRs have limited phylogenetic expression, where homologues are detected only in species of the same order and in some cases only within a particular species. Using phylogeny as the basis, we introduced an IR classification system, where tuning IRs were labeled as “protostome conserved,” “arthropod conserved,” “pancrustacean conserved,” “crustacean conserved,” and “decapod conserved.” This classification scheme can be expanded to other clades across protostomes. Although we performed a search for homologues of IRs across non-hexapod arthropods, it was by no means exhaustive. A broader and more thorough phylogenetic examination of IRs across protostomes would reveal lineages of conserved IRs and possibly give more insight into their function and evolutionary history across species. From our data, there appear to be candidate crustacean conserved tuning IRs (e.g. IR1020, Fig 5), decapod conserved IRs (e.g. IR1001, IR1018, IR1020, IR1021, IR1029), and arthropod conserved IRs (e.g. IR1039, IR21a, IR40a, IR75). Other tuning IRs appear to be insect conserved (e.g. IR31a, IR60a, IR64a) [3, 10, 19]. Finally, species-specific IRs have been found in crustaceans as well as insects [3, 11, 19, 87]. Although the chemical specificity of tuning IRs has been described for many IRs in *Drosophila* and other insect species [88], there have been no functional studies of IRs in crustaceans. So, while one might assume that a given IR expressed in different species confers the same response specificity, we do not have any data at this time to verify these assumptions. One particular case in point, IR40a, has been shown to mediate hygrosensation in fruit flies. Not only are there homologues to IR40a in all four aquatic decapods that we examined, but there are multiple homologues to IR40a in each species. If it is true that a given IR retains its specificity across species, then the nature of that specificity awaits future study.

How does the total number of IRs in these decapod crustaceans compare to other crustaceans and insects? Interestingly, *Daphnia pulex* has at least 210 types of chemoreceptor proteins in its genome– 154 IRs and 56 functional GRs–but where in the body those are expressed is not known. This is a relatively high number of chemoreceptor proteins, despite *Daphnia*’s highly reduced olfactory system. The copepod *Eurytempora affinis* has at least 83 types of chemoreceptor proteins– 22 IRs and 61 GRs [10, 18]. The antennular chemoreceptor proteins in two species of hermit crabs have been studied, and they have up to 29 IRs and no ORs or GRs per species [18]. Insect species have been described as typically having 20 to 150 IRs [3], but they also have ORs and GRs often in numbers equal to the IRs. For example, *D*. *melanogaster* has ca. 60 IRs and 68 GRs, plus ca. 60 ORs, for a total of nearly 200 functional chemoreceptor proteins. Thus, these four decapod crustaceans appear to have a relatively high number of IRs compared to many other crustaceans, and this high diversity plus the heterotetrameric organization of IRs allows many combinations and thus diverse chemical response spectra. We do not yet know the number of different IRs expressed in individual receptor neurons, but multiple types of heterotetramers are theoretically possible in single cells.

The levels of expression of IRs differ between the two chemosensory organs, the LF and dactyl. For most IRs, the expression level is higher (log_2_ fold change ≥ 1.5, or ≤ -1.5) in one chemosensory organ than the other, and in almost all of these cases of disproportionate expression levels, the LF has higher expression than the dactyl. What is the function of these “LF enriched/specific IRs” vs. the “LF-dactyl shared IRs”? This is not known since functional expression studies have not yet been done. However, it is tempting to speculate that the IRs shared by the LF and dactyl are sensitive to the compounds that both appendages are known to detect–food-related compounds such as amino acids, amines, nucleotides, nucleosides [89]. On the other hand, the LF-specific IRs might be expected to detect chemicals that only the LF senses–and these are pheromones, including sex, social, and alarm chemical signals and cues [89]. The legs are rarely described as being involved in the detection of pheromones [89], though there are exceptions [90]. This correlates with a lack of dactyl rich expression of IRs. In any case, the trend toward greater expression of IRs in LF vs. dactyl may be due to their expression in higher numbers in olfaction (i.e. in OSNs, which are found only in LF) than in distributed chemoreception (i.e. in CSNs, which are found in LF and dactyl).

### Chemoreception beyond IRs

The IRs appear to be the major chemoreceptor proteins in most crustaceans, but others are likely to contribute to their total chemosensory repertoire. Most likely are TRP channels and to some extent GRs.

#### TRP channels

Previously, we found in *P*. *argus* members of every sub-family of the two groups of TRP channels, including those that are known to be chemoreceptors in other species including mammals, insects, and nematodes. In this study, we also found homologues of all these TRP channels in the other three decapod species. This includes the four subfamilies of TRP channels known to have chemosensory functions in other species: TRPA, including those related to TRPA1, painless, TRPA5, pyrexia, and waterwitch of insects. Similar to *P*. *argus*, there were no direct homologues to pyrexia or waterwitch with high branch support. Instead, there was a homologue in each decapod that clustered with the clade containing both pyrexia and waterwitch, and one additional sequence in *H*. *americanus* that clustered more closely with the pyrexia/waterwitch clade with low bootstrap support (Fig 8); TRPV, including those related to OSM-9 of *C*. *elegans* and Nanchung and Inactive of *D*. *melanogaster*; TRPC channels; and TRPM, including those related to insects and mammalians, and these seem to be particularly expanded in the crustaceans. Decapods appear to have an additional TRPM channel compared to insects. This additional TRPM channel (TRPMc) that was found in *P*. *argus*, *H*. *americanus*, and *P*. *clarkii*, clearly clusters away from the TRPM channel homologue found in insects. While *Daphnia* also has multiple TRPM channels, all of these cluster closer to the insect TRPM channel. We can only speculate about the function of these crustacean TRP channels, as sequence homology does not necessarily signify functional homology for TRP channels. However, it is reasonable to speculate that several of the crustacean TRP channels may function as chemical sensors, due to the multimodal nature and known chemo-sensitivity of TRP channels across animals.

#### GRs and GRLs

Using multiple sequence alignment of GRs and InterPro domain search, we confirmed our prior identification of one GRL in *P*. *argus*, and extended this by finding GRLs in the other three decapod species. These were found in both the LF and dactyl transcriptomes. GRs and/or GRLs have been found in other crustacean species, usually in low numbers [10, 11]. However, a recent paper by Poynton et al. (2018) [18] provides new insight into the potential importance of GRs in crustaceans. They annotated the GR gene family of *Hyalella azteca*, generated improved models of the genome assemblies for two crustacean species, and showed considerable expression of GRs in these species. The amphipod *H*. *azteca*, the branchiopod *Daphnia pulex*, and the copepod *Eurytempora affinis* have 155 (of which 41 are pseudogenes), 59 (3 pseudogenes), and 67 (6 pseudogenes) GRs respectively. Among crustaceans, it is not known whether GRLs are expressed in chemosensory cells, and no functional studies have been done. Our results show that GRLs also exist in the brain (at least for *H*. *americanus*), and GRLs have also been found in the transcriptome of the Y-organ, an endocrine gland, in the decapod crustacean *Gecarcinus lateralis* (blackback land crab) [91]. There is some evidence in species other than crustaceans that the GRLs may play roles in development [9], and any role that GRLs may have in chemoreception is speculative.

#### Others?

Beyond IRs, TRP channels, and possibly GRLs, other classes of chemoreceptor proteins have been identified in arthropods and other protostomes. One major class is the ORs, which appear to have evolved from GRs and to date have been identified only in insects [4–6]. We did not find any evidence for ORs in crustaceans in our work. Another class of chemoreceptor proteins are GPCRs, which have been shown or suggested to function as chemoreceptor proteins in some protostomes [27–30]. We found many rhodopsin-like GPCRs in our decapod transcriptomes, but most could be identified as homologues of known classes of GPCRs that are not mammalian ORs, and to date we have not found expanded families of orphaned GPCRs as might be expected if they function as chemoreceptor proteins (M. Rump, M. Kozma, and C. Derby unpublished results). Epithelial sodium channels (ENaC) are a class of ionotropic receptors that have been shown to be used by *Drosophila* for detecting salt [92], water [93, 94], and pheromones [95–97], in at least one case by activating downstream of IRs, ORs, or GRs in a calcium-dependent amplification step [25]. Though we found ENaCs in all four decapod transcriptomes, we did not find homologues of ppk23 and ppk28, the chemosensory *pickpocket* genes that are ENaC homologues in insects. Clearly, more analysis is necessary in order to fully evaluate the role of GPCRs, ENaCs, and other classes of receptor proteins in chemical sensing in crustaceans.

### Olfactory logic in decapod crustaceans

Given the heterotetrameric combinatorial nature of IRs and that there are over 100 co-receptor and tuning 100 IR units in the chemosensory organs of each of these four decapod species, and given the other candidate chemoreceptor proteins in these tissues including TRP channels, GPCRs, and ENaCs expressed, the breadth and scope of receptor molecules in these systems is potentially very large. To understand the olfactory logic in decapod crustaceans, one must know the patterns of expression of these receptor molecules in single OSNs, as well as the central projections of OSNs with defined expression patterns. For example, the antennule of *P*. *argus* and *H*. *americanus* has *ca*. 300,000 OSNs, and those OSNs project into *ca*. 1,200 glomeruli in the olfactory lobe [98]. Electrophysiological studies in lobsters suggest potentially dozens to hundreds of different physiological classes of chemosensory neurons [44, 99, 100], which might lead to the expectation of a high diversity of receptor expression patterns in the population of OSNs. This scenario raises the possibility of an olfactory logic in decapod crustaceans that is significantly different than in insects or mammals, in which the ratio of the number of types of receptor molecules to glomeruli is often *ca*. 1:1, with some exceptions [101–103]. To explore the olfactory logic in crustaceans, we need to perform single cell transcriptomics on hundreds of OSNs whose chemical sensitivities are defined, then classify these cells based on their receptor expression and physiological response profiles, and describe their patterns of innervation in the olfactory lobe.

## Conclusions

Decapod crustaceans have hundreds of candidate chemoreceptor proteins in their olfactory and distributed chemoreception systems. IRs are certainly major chemoreceptor molecules in crustaceans, though there is almost no functional analysis of their roles. More work is necessary to determine the chemical sensitivity of different families of IRs or specific IRs. TRP channels of decapod crustaceans are likely to include some chemoreceptor proteins, as crustacean homologues of TRP channels with chemosensory functions in other species are identified. Still, experimental evidence for their roles in chemoreception are completely lacking. The role of GRs and GRLs in crustaceans in general is more difficult to evaluate, in part because the extent of their expression seems to vary tremendously across the clades of crustaceans, from more than 100 in branchiopods and amphipods to one or few in decapods. Other classes of receptor proteins, including suspected (GPCRs and ENaCs) and others not identified, also need to be further considered as possible candidates. Future studies should use single cell transcriptomics to understand the combinatorial expression patterns of chemoreceptor proteins in single chemosensory neurons, examine function of receptor proteins by examining receptor expression in single cells whose chemical sensitivities are defined, and by experimentally determining the chemical sensitivities of specific receptor proteins through heterologous expression of combinations of receptor molecules and/or through regulation of receptor expression levels in chemosensory neurons.

## Supporting information

S1 Fig Radial tree configuration of phylogenetic analysis of iGluRs and IRs from decapod crustaceans (otherwise represented in Fig 4). (TIF)

S1 Table Transcript and protein coding gene counts generated from EVG pipeline. (XLSX)

S2 Table BUSCO output. (XLSX)

S3 Table Decapod iGluRs. (XLSX)

S4 Table RSEM counts matrix for *P*. *argus* of LF, dactyl, and brain. (CSV)

S5 Table RSEM counts matrix for *H*. *americanus* of LF, dactyl, and brain. (CSV)

S6 Table RSEM counts matrix for *P*. *clarkii* of LF, dactyl, and brain. (CSV)

S7 Table RSEM counts matrix for *C*. *sapidus* of LF and dactyl. (CSV)

S8 Table DESeq2 analyses of LF vs. dactyl in *P*. *argus*. (CSV)

S9 Table DESeq2 analyses of LF vs. dactyl in *H*. *americanus*. (CSV)

S10 Table DESeq2 analyses of LF vs. dactyl in *P*. *clarkii*. (CSV)

S11 Table DESeq2 analyses of LF vs. dactyl in *C*. *sapidus*. (CSV)

S12 Table Sequence IDs and Accession Numbers. (XLSX)

S1 File DESeq2 analysis—R script. (R)

S2 File All sequences containing PF00063 in *P*. *argus*. (FASTA)

S3 File All sequences containing PF10613 in *P*. *argus*. (FASTA)

S4 File All sequences containing PF00063 in *H*. *americanus*. (FASTA)

S5 File All sequences containing PF10613 in *H*. *americanus*. (FASTA)

S6 File All sequences containing PF00063 in *P*. *clarkii*. (FASTA)

S7 File All sequences containing PF10613 in *P*. *clarkii*. (FASTA)

S8 File All sequences containing PF00063 in *C*. *sapidus*. (FASTA)

S9 File All sequences containing PF10613 in *C*. *sapidus*. (FASTA)

S10 File Sequences for Fig 4. (FASTA)

S11 File Mafft aligned sequences for Fig 4. (FASTA)

S12 File Trimmed-Mafft aligned sequences for Fig 4. (FASTA)

S13 File Sequences for Fig 5. (FASTA)

S14 File Mafft aligned sequences for Fig 5. (FASTA)

S15 File Trimmed-Mafft aligned sequences for Fig 5. (FASTA)

S16 File Sequences for Fig 8. (FASTA)

S17 File Mafft aligned sequences for Fig 8. (FASTA)

S18 File Trimmed-Mafft aligned sequences for Fig 8. (FASTA)

S19 File Sequences for Fig 9. (FASTA)

S20 File Mafft aligned sequences for Fig 9. (FASTA)

S21 File Trimmed-Mafft aligned sequences for Fig 9. (FASTA)
