# Supplementary figures and images for: Comparison of transcriptomes from two chemosensory organs in four decapod crustaceans reveals hundreds of candidate chemoreceptor proteins
Source: PLoS One. 2020 Mar 12;15(3):e0230266. doi: 10.1371/journal.pone.0230266 (PMC7067487; doi:10.1371/journal.pone.0230266)

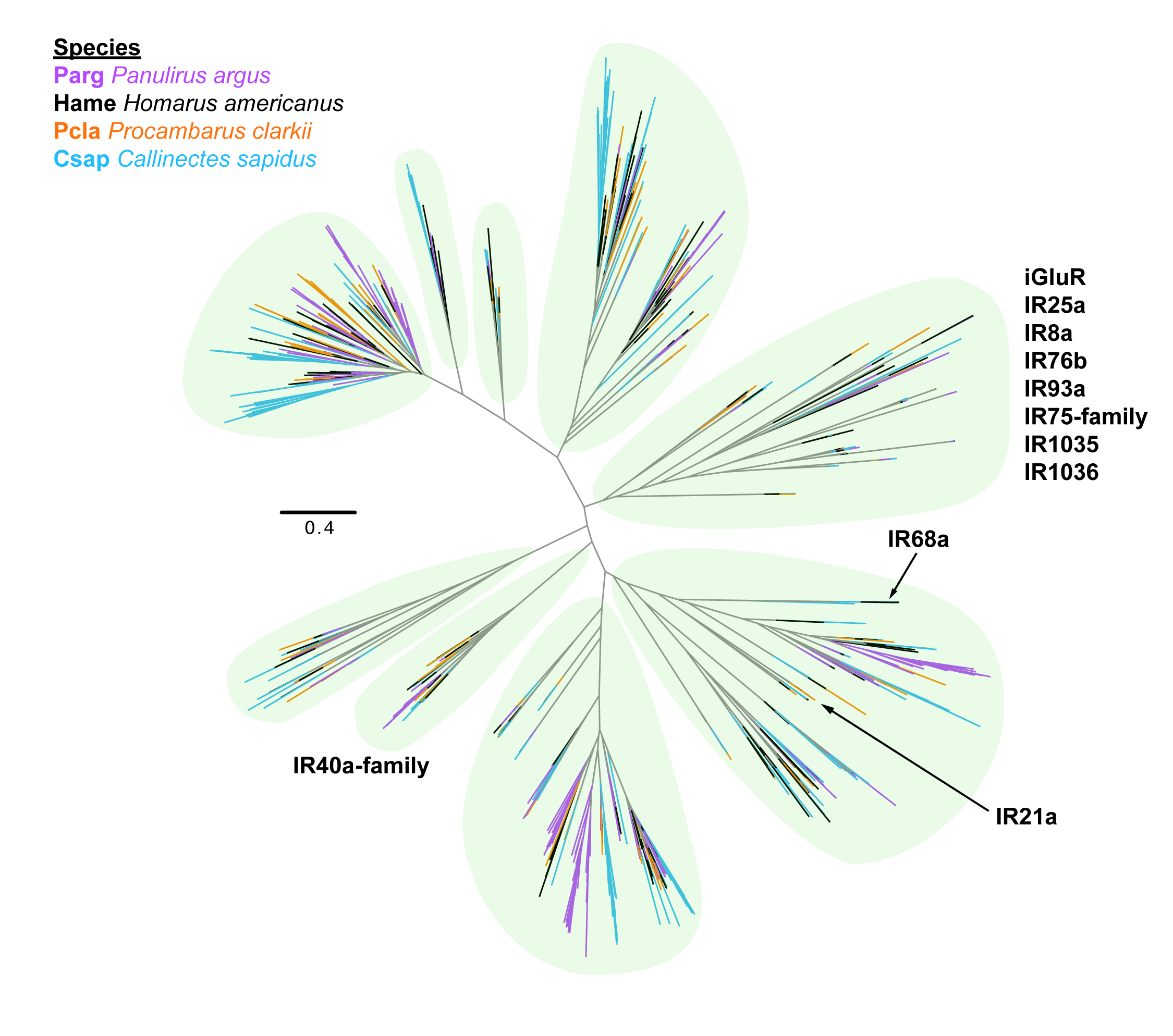

Supplement: S1 Fig — (TIF) [file pone.0230266.s001.tif]
